# Author Correction: Accelerating discovery of bioactive ligands with pharmacophore-informed generative models

**DOI:** 10.1038/s41467-025-58701-w

**Published:** 2025-04-07

**Authors:** Weixin Xie, Jianhang Zhang, Qin Xie, Chaojun Gong, Yuhao Ren, Jin Xie, Qi Sun, Youjun Xu, Luhua Lai, Jianfeng Pei

**Affiliations:** 1 Center for Quantitative Biology, Academy for Advanced Interdisciplinary Studies, Peking University, Beijing, China; 2 Infinite Intelligence Pharma, Beijing, China; 3 BNLMS, Peking-Tsinghua Center for Life Sciences at the College of Chemistry and Molecular Engineering, Peking University, Beijing, China; 4 Peking University Chengdu Academy for Advanced Interdisciplinary Biotechnologies, Chengdu, China; 5 Research Unit of Drug Design Method, Chinese Academy of Medical Sciences, Beijing, China

**Keywords:** Cheminformatics, Drug discovery and development, Machine learning

Correction to: *Nature Communications* 10.1038/s41467-025-56349-0, published online 10 March 2025

In this article the funding from “Anhui’s Plans for Major Provincial Science & Technology Projects (Grant 202303a07020009)” was omitted. In addition, the following information was missing from the Acknowledgement section: ‘We thank Dr. Guangwei He for insightful discussions on experimental design and are grateful to his team at HIPI for their support during experiments. We also acknowledge Dr. Chu, Dr. Xu, and Minghan He for their assistance with statistical analysis and design’. The original article has been corrected.

