# Supplementary material for: Accelerating discovery of bioactive ligands with pharmacophore-informed generative models
Source: Nat Commun. 2025 Mar 10;16:2391. doi: 10.1038/s41467-025-56349-0 (PMC11894060; doi:10.1038/s41467-025-56349-0)
Supplement: Supplementary file 1 — Supplementary Information [file 41467_2025_56349_MOESM1_ESM.pdf]

Supplementary Information for Accelerating Discovery of Bioactive Ligands with Pharmacophore Informed Generative Models

Weixin Xie[1,†], **Jianhang Zhang**[2,†], **Qin Xie**[2], **Chaojun Gong**[2], **Yuhao Ren**[3], **Jin Xie**[1], **Qi Sun**[3,4,5], **Youjun Xu**[2,*], **Luhua Lai**[1,3,4,5,*], and **Jianfeng Pei**[1,5,*]

[1]Center for Quantitative Biology, Academy for Advanced Interdisciplinary Studies, Peking University, Beijing 100871, China
[2]Infinite Intelligence Pharma, Beijing 100012, China
[3]BNLMS, Peking-Tsinghua Center for Life Sciences at the College of Chemistry and Molecular Engineering, Peking University, Beijing 100871, China
[4]Peking University Chengdu Academy for Advanced Interdisciplinary Biotechnologies, Chengdu 610200, Sichuan, China
[5]Research Unit of Drug Design Method, Chinese Academy of Medical Sciences (2021RU014), Beijing 100871, China

[†]Equal contribution
[*]Corresponding authors: xuyj@iipharma.cn, lhlai@pku.edu.cn, jfpei@pku.edu.cn

Supplementary Notes

Pharmacophore fingerprints as fuzzy and interpretable representations

We generated 72-bit fingerprints and analyzed the pharmacophoric features and their two-point combinations for compounds in the GuacaMol dataset[1], as depicted in Supplementary Figure 12. The pharmacophoric features considered in our work were hydrogen bond acceptor (ACC), hydrogen bond donor (DON), aromatic (ARO), negative ionizable (NEGI), positive ionizable (POSI), hydrophobic (HYD), lumped hydrophobic (LHYD), and Zn-ion binder (ZB), which were defined following RDKit's definitions. One common observation is that hydrogen bond acceptors and hydrophobic groups are the most frequently occurring features, whereas negative positive ionizable groups and Zn-ion binders are the least frequent (Supplementary Figure 12a). Since the GuacaMol dataset mainly comprises drug-like and synthesizable compounds from the ChEMBL database,[1, 2] the relative numbers and ratio of the ACCs and DONs closely match the recent trends in developability molecular properties[3]. The results in Supplementary Figure 12b demonstrated that the proportions of F1-F2-0 bits (feature F1 and F2 separated by a low distance bin) were often less than those of F1-F2-1 bits (F1 and F2 separated by a high distance bin). This suggests that bits with a high distance bin are preferred feature combinations for druglike compound libraries, such as the GuacaMol dataset, particularly for ACC-ACC-0/1, ACC-POSI-0/1, ARO-ARO-0/1, ARO-LHYD-0/1, DON-DON-0/1, DON-LHYD-0/1, DON-POSI-0/1, HYD-LHYD-0/1, and LHYD-LHYD-0/1. However, ionizable pairings had a small percentage in the GuacaMol database, which may affect TransPharmer's capacity to generate ionizable molecules.

In TransPharmer, the pharmacophore fingerprint was designed to emphasize the interplay between pharmacophores rather than focusing on individual pharmacophores, which allows for a better understanding of the correlation between individual pharmacophoric features and bioactivity. Similar concepts such as triplet pharmacophoric characteristics have already been employed in pharmacophore-based virtual screening [4]. The t-SNE plots (Supplementary Figure 13) of the tested compounds on DRD2 demonstrate the potential of pharmacophore fingerprints we used to discriminate active and inactive compounds. Consequently, these pharmacophore fingerprints are likely to offer valuable guidance for the generation of bioactive molecules.

In order to exhibit the intuitive differences between other molecular representations (i.e. ECFP[5] or reduced graph[6, 7]) and the pharmacophore fingerprints we used, we tried to find pairs of compounds that are both pharmacophorically similar and structurally dissimilar among the 7939 DRD2 (Dopamine Receptor D2) actives recorded in the ExCAPE-DB[8]. The S_{pharma} and structural similarity S_{struct} are computed using the pharmacophore fingerprints and the Morgan circle fingerprints of radius 2, and the details are explained in the section of Methods. We found that over 0.3 million molecular pairs have a $S_{\text{pharma}} > 0.9$ and a $S_{\text{struct}} < 0.3$. Even when a $S_{\text{pharma}} > 0.99$ and a $S_{\text{struct}} < 0.1$ are required, there are still 943 molecular pairs left. When a fine-grained (3-bin) pharmacophore fingerprint with 108 bits is used, there are fewer molecular pairs that satisfy the requirement. There are only 4 molecular pairs left under the requirement of a $S_{\text{pharma}} > 0.99$ and a $S_{\text{struct}} < 0.1$. The example pairs found of each case are shown in Supplementary Figure 14. These results indicate that the pharmacophore fingerprint appears to be one fuzzier representation than the reduced graph representation suggested by Pogány et al.[9], due to those compound pairs are unlikely to be covered by a meaningful reduced graph. Bit collisions that occur during fingerprint construction would increase the probabilities of fuzziness. Actually, such fuzzy characteristic of pharmacophore fingerprint may make it useful for finding novel and diverse candidate compounds while preserving similar pharmacophores.

Benchmarking the unconditional TransPharmer and other evaluations

To compare with other unconditional generative models, we trained unconditional versions of TransPharmer. The distribution learning performance, benchmarked by the GuacaMol benchmarking suite[1], demonstrates that TransPharmer is competitive with established generative models, achieving state-of-the-art performance in overall metrics (Supplementary Table 10). Additionally, the unconditional TransPharmer re-trained on the MOSES dataset[10] ranks among the top two in six out of fifteen metrics compared to other models

(Supplementary Table 11). These results underscore TransPharmer's capability to model the chemical space accurately.

We also assessed our conditional model's performance on rediscovering six marketed drugs using the GuacaMol goal-directed benchmarks. We exhaustively sampled 10,000 valid molecules using the 72-bit pharmacophore fingerprints of six target drugs as queries. As indicated in Supplementary Table 12, our model successfully rediscovered five out of six drug molecules ($S_{\text{struct}} = 1$). Unlike other models that require iterative sampling and evaluations, our model utilized a single fuzzy fingerprint as a condition to recall each drug directly.

For Troglitazone, we observed difficulties in recalling the complete molecule due to the pharmacophoric insufficiency of encoding tetramethylchroman, as depicted in Supplementary Figure 16. Implementing a constrained strategy facilitated the recall of this molecule, suggesting that the model focuses on generating scaffolds with similar pharmacophore features and may neglect side chains and ring formations with fewer pharmacophore features. In Supplementary Figure 17, we presented the distributions of molecular structures and related similar (> 0.7) and dissimilar (< 0.5) samples for Celecoxib. Several plausible topological modifications, such as sidechain replacement, heterocyclic substitution, macrocyclic substitution, and ring replacement, were evident in Supplementary Figure 17b.

To assess the fingerprint conformance of our model, we generated 1,000 molecules for each of 1,000 samples from the GuacaMol test set under three conditions: 72-bit, 108-bit, and 1,032-bit fingerprints. We computed the fingerprint deviation of the generated molecules from the query molecules. The results summarized in Supplementary Figure 15a indicate that the 72-bit model achieved the highest conformance. Beyond fingerprint dimensionality, one possible explanation is that the 72-bit fingerprint represents one of the most frequently occurring coarse-grained structural feature pairs. The model learns these high-frequency bits effortlessly but struggles with low-frequency bits. In Supplementary Figure 15b and c, we utilized the TMap toolbox to visualize the tree-like distributions based on S_{pharma} and S_{struct} (see Section "Evaluation metrics" in the main text). The differentiated patterns of both distributions imply that our model employs these pharmacophore fingerprints to explore a focused chemical space extending beyond structural similarity.

To evaluate the quality of the generated molecules, we produced 1,000 molecules for each of 1,000 randomly chosen seeds from the test set. The plotted comparable distributions concerning six typical molecular properties (LogP, HBA, HBD, RotB, QED, and SAS) between the generated molecules and the test set are shown in Supplementary Figure 15d-i. These matched properties demonstrate that our model can perform effective sampling within a reasonable chemical space.

Further evaluation of and comparison with PGMG

We noted that PGMG was designed to generate compounds that align with a given pharmacophore hypothesis, which typically includes three to seven pharmacophoric features, a subset of the features extracted from the reference compound. In contrast, TransPharmer considers all pharmacophoric features present in the reference compound. We speculated that PGMG might struggle to generate compounds with similar global properties, such as molecular weight, to the reference compound when using incomplete pharmacophoric information, potentially leading to reduced sampling efficiency.

To evaluate this hypothesis, we tested PGMG on the same test set described in the main text, with the maximum number of input features in the pharmacophore hypothesis limited to three or eight. If the reference compound contained fewer than three or eight pharmacophoric features, all features were included. These settings represent the two extremes of the default input feature range (three to seven) in PGMG. We labeled PGMG taking a maximum of three input features as "PGMG-max3pp" and PGMG taking a maximum of eight input features as "PGMG-max8pp". Additionally, given that PGMG uses 3D pharmacophore hypotheses, we evaluated its performance on reference compounds capable of adopting diverse 3D conformations. We curated a subset of 114 molecules exhibiting flexible conformations from the original test set of 300 target compounds. A molecule is considered to have a flexible conformation if, after using RDKit's ETKDG to generate ten independent conformations, at least one conformation has an RMSD over 2 Å compared to the first conformation. PGMG evaluated on these 114 molecules is labeled as "PGMG

(114 cases, embed1)" for molecules adopting the first conformation and "PGMG (114 cases, embed2)" for molecules adopting the conformation with RMSD > 2 Å.

The evaluation results are shown in Supplementary Table 2. The D_{count} and S_{pharma} metrics did not show significant improvement or deterioration for PGMG under different settings or evaluation sets. However, "PGMG-max3pp" tended to generate compounds with smaller sizes compared to the reference compounds, whereas "PGMG-max8pp" tended to generate compounds with larger sizes. This significant deviation in molecular sizes was also observed in the evaluation set of 114 target compounds with flexible conformations. These findings indicate that PGMG may have difficulty in accurately inferring the size of the reference compound based on incomplete pharmacophoric information. This sensitivity to the number of input features necessitates careful selection of an appropriate pharmacophore hypothesis to ensure PGMG produces compounds with similar molecular properties. In contrast, TransPharmer can efficiently generate molecules with high pharmacophoric similarity and sizes comparable to the reference compounds.

Comparison with REINVENT

REINVENT is a generative model that leverages reinforcement learning to bias molecule generation towards desired targets[11, 12], presenting an alternative to the conditional generative models benchmarked in this study. We compared our model with REINVENT, which was rewarded based on pharmacophore similarity, S_{pharma} (72-bit) (calculated using Tanimoto similarity on 72-bit pharmacophore fingerprints rather than ErG fingerprints). Based on REINVENT v3.2, we modified the model to accept user-defined scoring components. The REINVENT model was initially pretrained on the ChEMBLv32 database for 20 epochs, as recommended, to create a prior model. Subsequently, new agents were built on this prior, guided by the pharmacophore similarity to each molecule from the same test set used to benchmark other pharmacophore-based conditional generative models. A total of 300 REINVENT agents were obtained through a unified reinforcement learning process that goes through 1,000 steps. Finally, agents from the final step were sampled 1,000 times for evaluation, and the results were compared with those generated by TransPharmer-72bit. The results are summarized in Supplementary Table 13. TransPharmer outperformed REINVENT by generating more compounds with higher pharmacophore similarity and a lower deviation in the number of pharmacophore features.

We also applied it to the case study of designing PLK1 inhibitors, using Onvansertib as the target compound, as demonstration. The reinforcement learning curve is shown in Supplementary Figure 18. REINVENT required approximately 53 minutes (1,000 steps) to converge for this single goal, whereas TransPharmer, once trained, was able to generate the same number of molecules within 1 minute. The molecules generated by TransPharmer better aligned with the target topological pharmacophore, exhibiting higher S_{pharma} and S_{pharma} (72bit) scores, and a lower D_{count} (Supplementary Table 14). The low efficiency of REINVENT was expected, as reinforcement learning can take long time to explore the chemical space and the model may sometimes fall into suboptimal solutions. In contrast, conditional generative models like TransPharmer demonstrate greater efficiency in sampling molecules that satisfy the target goals.

Ablation study

We conducted an ablation study to evaluate the impact of pharmacophore fingerprints and their key features in guiding the exploration of relevant chemical subspaces. Two variants of TransPharmer were constructed for this purpose. The first variant utilized a reduced form of the pharmacophore fingerprint that omits the topological distance information to initiate molecule generation. The second variant employed another reduced version of the pharmacophore fingerprint, which omit the topological distance information and encode the occurrence of pharmacophore feature singlets instead of their combinations. Additionally, the unconditional TransPharmer, regarded as a baseline model without any pharmacophore guidance, was included for comparative analysis.

As described in the "Pharmacophore-Constrained Molecule Generation" section of the main text, these TransPharmer variants were trained on the GuacaMol dataset to perform both *de novo* molecule generation and scaffold elaboration tasks. For the *de novo* generation task, a total of 600 molecules were sampled from

scratch and used to calculate similarity scores and feature deviations. For the unconditional TransPharmer, the same generated set was duplicated for each conditioning compound in the test set. For the scaffold elaboration task, the same initial fragments were employed to initiate generation, but with reduced guidance of pharmacophore information. Similarity scores and feature deviations were obtained by comparing the generated molecules to their corresponding conditioning compounds. The results, summarized in Supplementary Table 15, demonstrate that pharmacophore fingerprints significantly enhance the generative model's ability to navigate towards the desired local chemical space. Furthermore, it is evident that the incorporation of topological distance information and feature combinations into the pharmacophore fingerprint substantially contributes to TransPharmer's overall performance. For the *de novo* generation task, removing the topological distance information decreased the pharmacophore similarity score from 0.50 to 0.38 and removing both the topological distance information and feature combinations further decreased it to 0.31. For the scaffold elaboration task, removing both the topological distance information and feature combinations decreased the pharmacophore similarity score from 0.70 to 0.55.

Molecular novelty assessment of the discovered hits

Within the ExCAPE-DB database, we searched for the most similar active ligands of PLK1 to IIP0942, IIP0943 and IIP0945, respectively, by computing the Tanimoto index using 2048-bit Morgan fingerprints with a radius of 2 (referred to as Morgan similarity). The most similar known inhibitors in ExCAPE-DB all feature different scaffolds from those of the designed compounds (Supplementary Figure 7).

Within the ChEMBL database, we queried for compounds demonstrating a similarity exceeding 0.4 to IIP0943, the most potent hit in this study. Among 229 compounds displaying a similarity, we retained 99 of them that shared the substructure of 4-phenoxypyrimidine present in IIP0943. These 99 compounds have a similarity below 0.59 to IIP0943 (Supplementary Figure 8b). They were manually inspected and further classified into three groups based on their common substructures (Supplementary Figure 8a). Representative compounds of each group were chosen, considering their potency, presence of documented literature or patent records in ChEMBL, and presence of co-crystallized complex PDB entries (Supplementary Figure 8c). It is noteworthy that BI-4464, the picked representative of group 4 and an inhibitor of the FAK kinase, shares similarity with IIP0943 in both chemical structure and binding mode within the ATP pocket. Given this results, we tested the inhibitory activities of the designed compounds against FAK for selectivity concerns.

We also searched for analogs of IIP0943 using SciFinder. By setting the similarity cutoff to 0.65 and eliminating items with more than one component and those lacking available references, we were left with 6,579 items. The compound identified as the most similar by SciFinder is shown in Supplementary Figure 9a. Subsequently, we downloaded the top 500 most similar compounds and computed their Morgan similarity to IIP0943. Supplementary Figure 9b illustrates the compound with the highest Morgan similarity. Given that both the compounds in Supplementary Figure 9a and b feature a distinct scaffold from the 4-(benzo[*b*]thiophen-7-yloxy)pyrimidine in IIP0943, which found no identical matches within the entire set of 500 compounds as well, our focus shifted to compounds containing a 4-phenoxypyrimidine moiety. The most similar compound within this subset is shown in Supplementary Figure 9c, with a Morgan similarity to IIP0943 of 0.56. Finally, we performed a direct search for compounds containing the 4-(benzo[*b*]thiophen-7-yloxy)pyrimidine moiety in SciFinder, with only one unrelated outcome (Supplementary Figure 9d).

Supplementary Figures

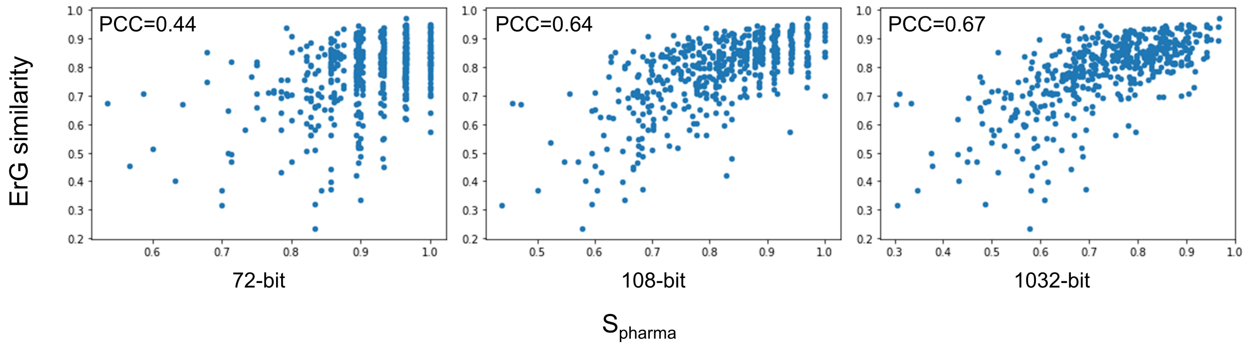

Supplementary Figure 1: Pearson correlation coefficients (PCC) between similarity scores computed with ErG fingerprints and those computed with 72-, 108- and 1032-bit pharmacophore fingerprints utilized in TransPharmer, respectively.

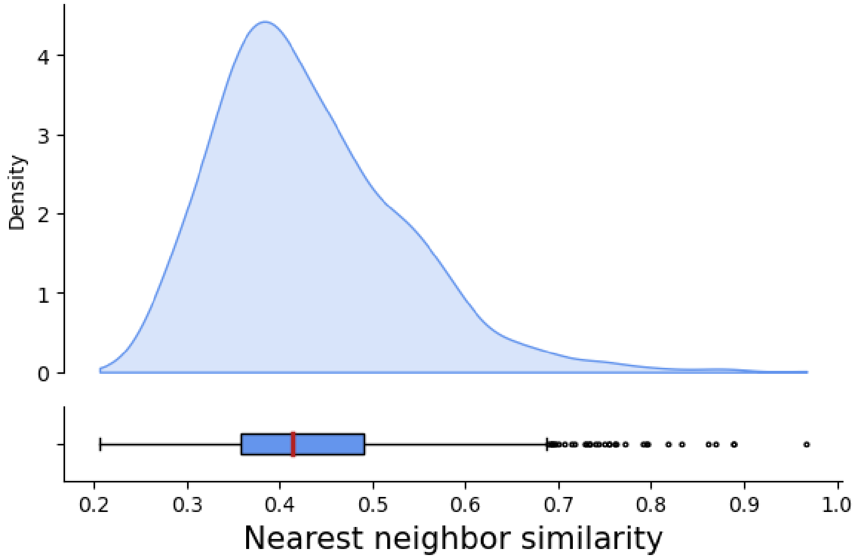

Supplementary Figure 2: The distribution of nearest neighbor similarity of the generated compounds to known DRD2 actives.

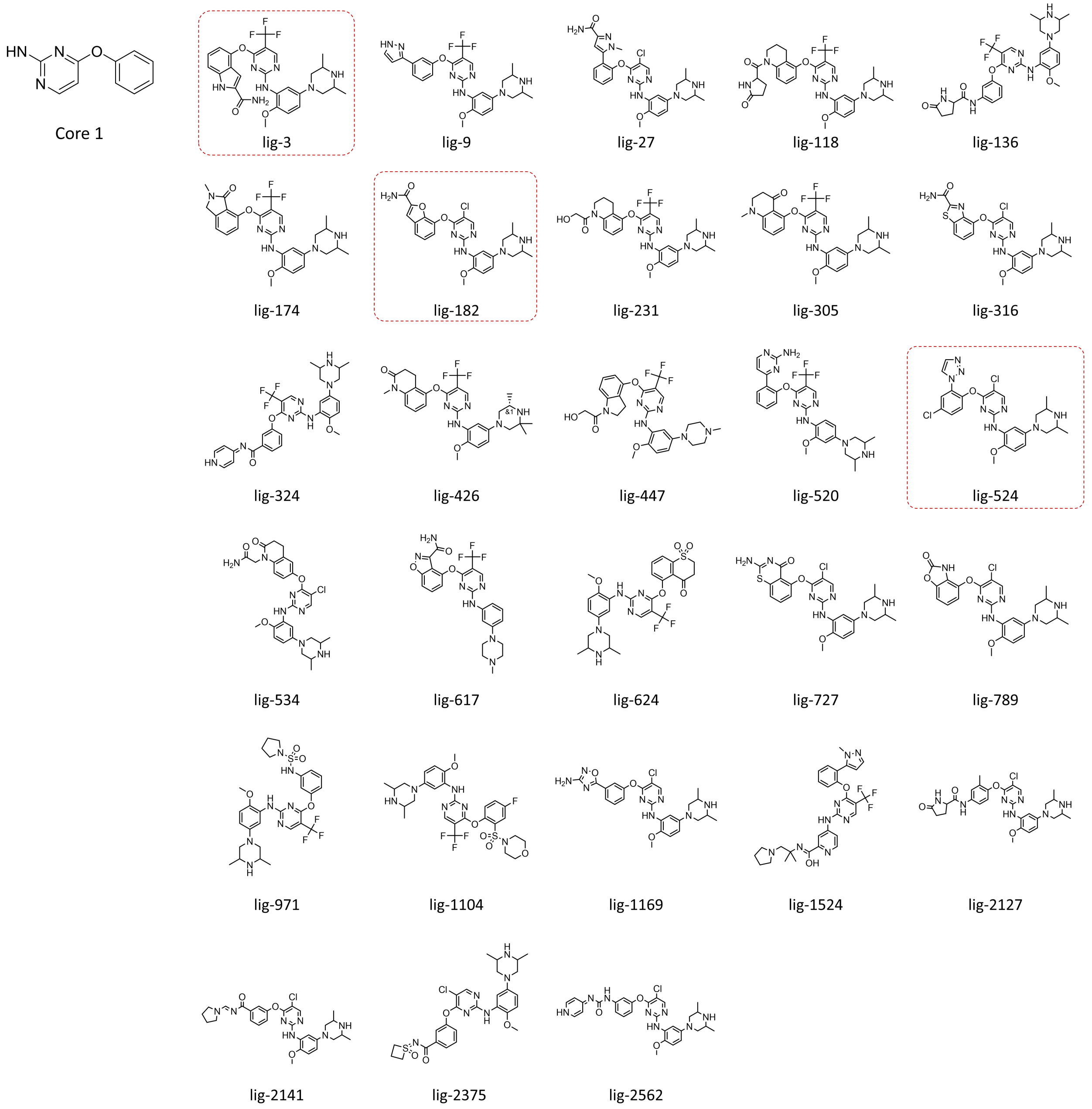

Supplementary Figure 3: The chemical structures of 42 hit compounds. Here are the 28 generated compounds (right) carrying core 1 (left), with a molecule ID below each structure. The dashed red squircles indicate the hit compounds selected for chemical synthesis and experimental testing.

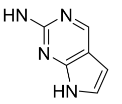

Core 2

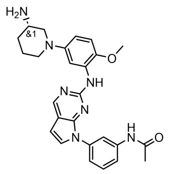

lig-21

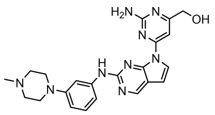

lig-121

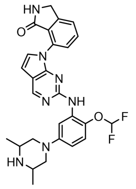

lig-325

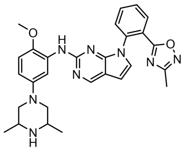

lig-886

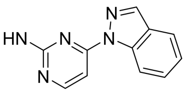

Core 3

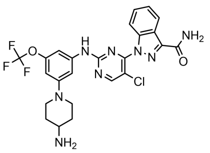

lig-603

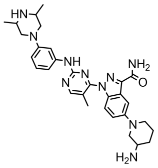

lig-648

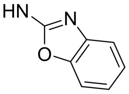

Core 4

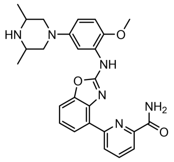

lig-522

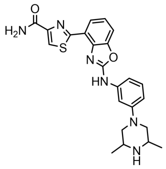

lig-1068

Other cores

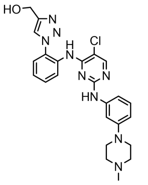

lig-26

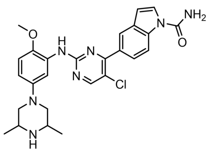

lig-575

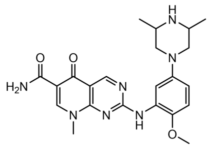

lig-669

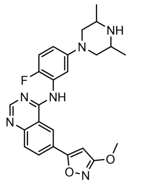

lig-891

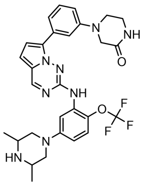

lig-962

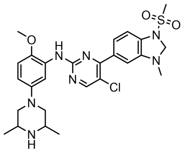

lig-1123

Supplementary Figure 4: The chemical structures of 42 hit compounds (continued). Here are the generated compounds (right) carrying core 2, 3 or 4, or alternative cores (left), with a molecule ID below each structure. The dashed red squircles indicate the hit compounds selected for chemical synthesis and experimental testing.

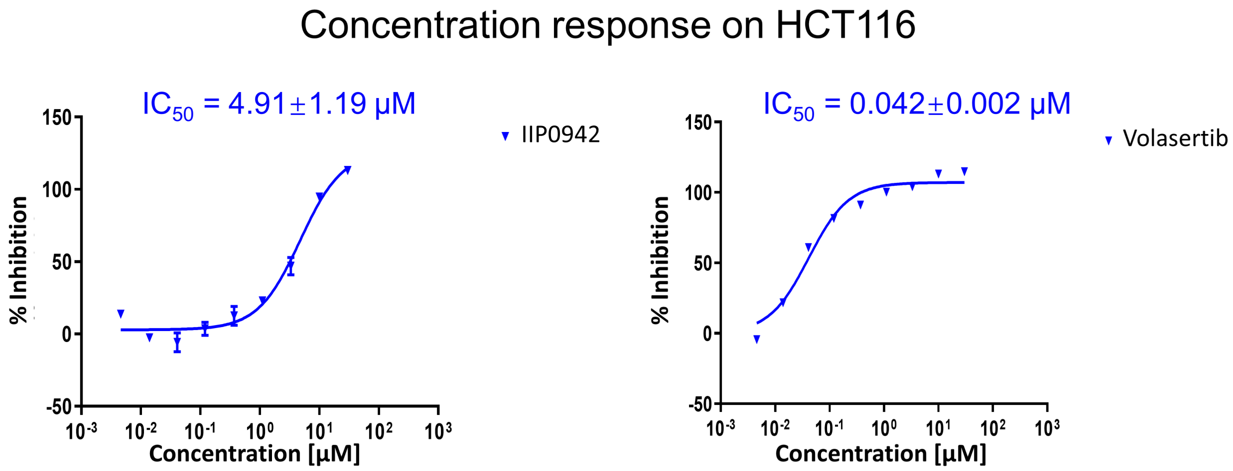

Supplementary Figure 5: Concentration-response curves of IIP0942 and Volasertib in the CellTiter-Glo assays on HCT116 cell lines, respectively. Data are presented as mean ± standard deviation (n=3 replicates).

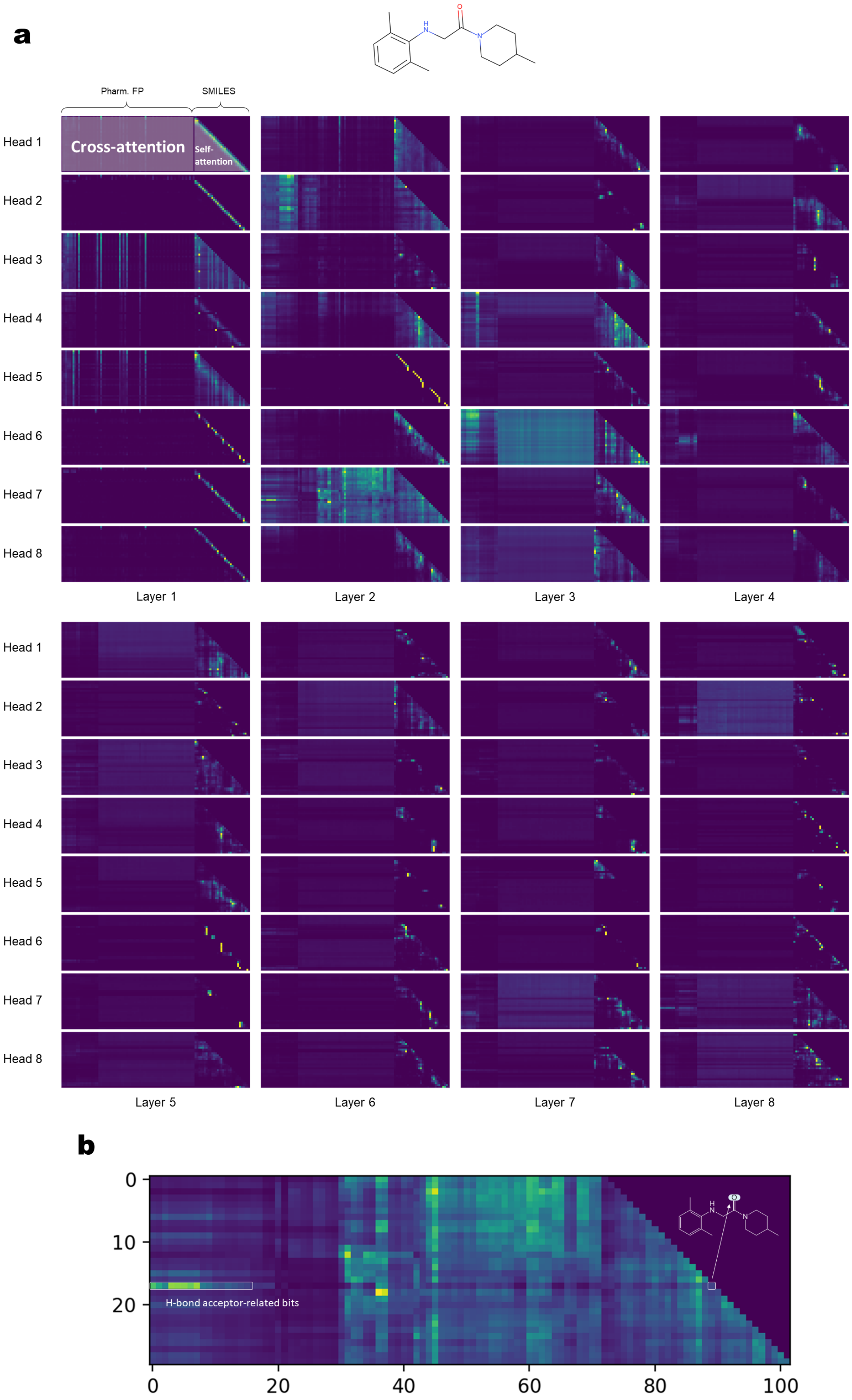

Supplementary Figure 6: The attention maps of an exemplar compound. (a) Attention maps across all transformer block and attention heads. (b) The attention map at the 7th head in the second layer.

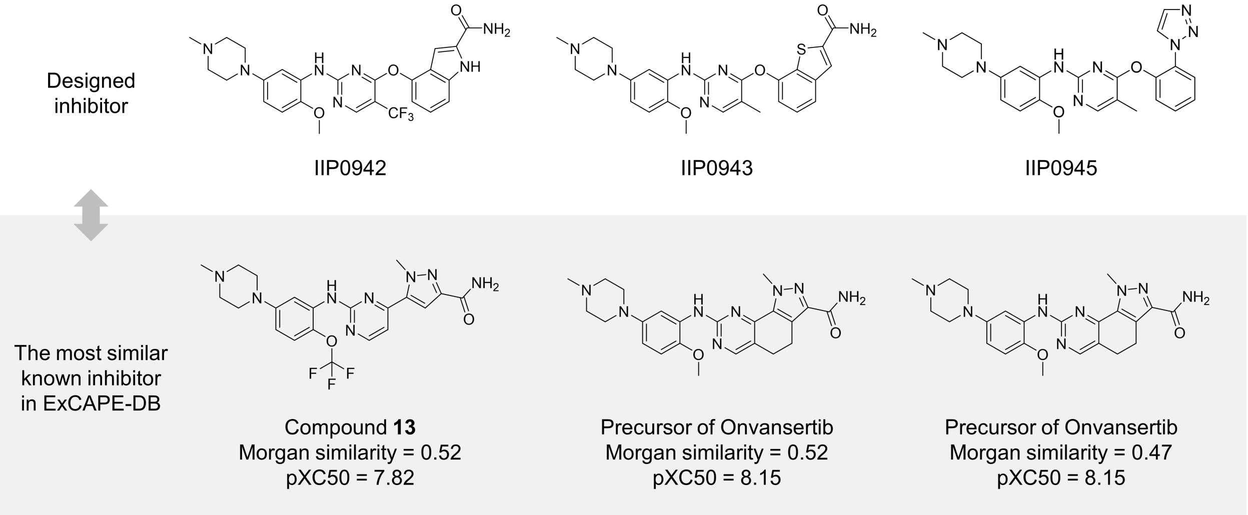

Supplementary Figure 7: Novelty assessment within the ExCAPE-DB database. Top: the chemical structures of active PLK1 inhibitors generated by TransPharmer. Bottom: the most similar known inhibitor in ExCAPE-DB, along with the computed similarity score corresponding to the generated compound and the associated pXC50 record (from left to right: compound **13**[13] and the precursor of Onvansertib[14]).

a

Substructure	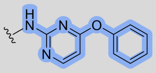	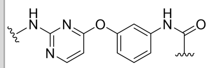	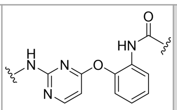	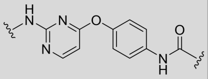	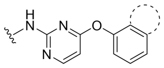
Group id	0	1	2	3	4
Count	99	63	13	7	16
Note	66 of them in TransPharmer's training set			Derivatives of group 1	Fused five-membered aliphatic ring

b

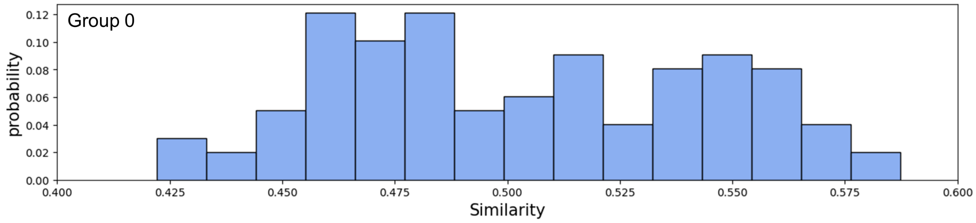

c

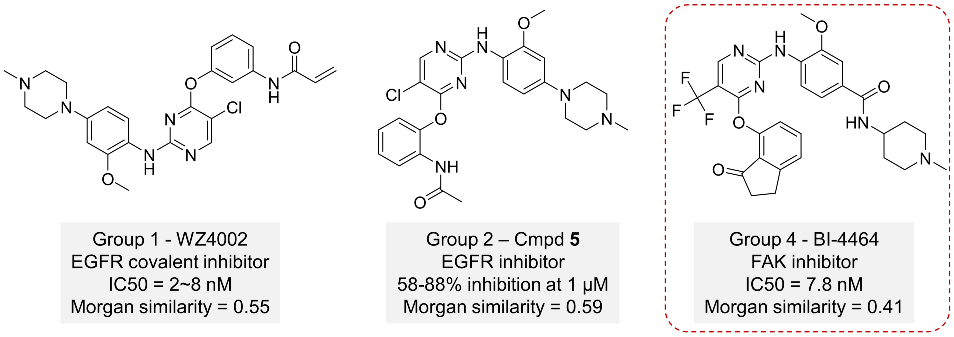

Supplementary Figure 8: Novelty assessment of IIP0943 within the ChEMBL database. (a) The categorization of the 99 compounds (group 0) in ChEMBL that carry the substructure of 4-phenoxypyrimidine and show similarity to IIP0943. (b) The Morgan similarity distribution for the 99 compounds in group 0. (c) Representative members of group 1, 2 and 4 are presented (from left to right: WZ4002[15], compound **5**[16] and BI-4464[17, 18]). The dashed red circle highlights BI-4464 as a FAK inhibitor. Compound **5** in group 2 is also the most similar structure to IIP0943 within those retrieved from ChEMBL.

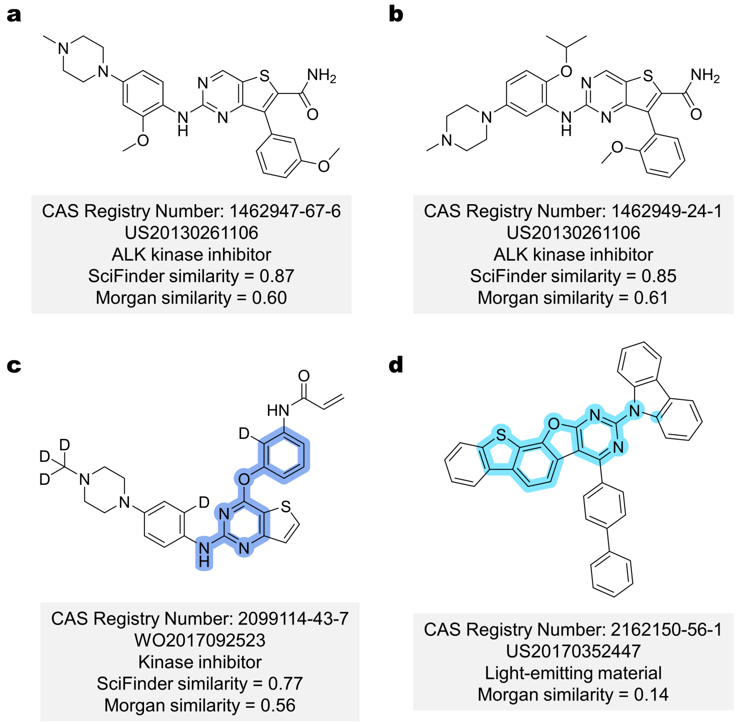

Supplementary Figure 9: Novelty assessment of IIP0943 using SciFinder. (a) The most similar compound identified by SciFinder. (b) The most similar compound within the top 500 SciFinder items in terms of Morgan similarity. (c) The most similar compound within the top 500 SciFinder items carrying a 4-phenoxypyrimidine moiety. (d) The only compound returned by SciFinder carrying a 4-(benzo[*b*]thiophen-7-yloxy)pyrimidine substructure.

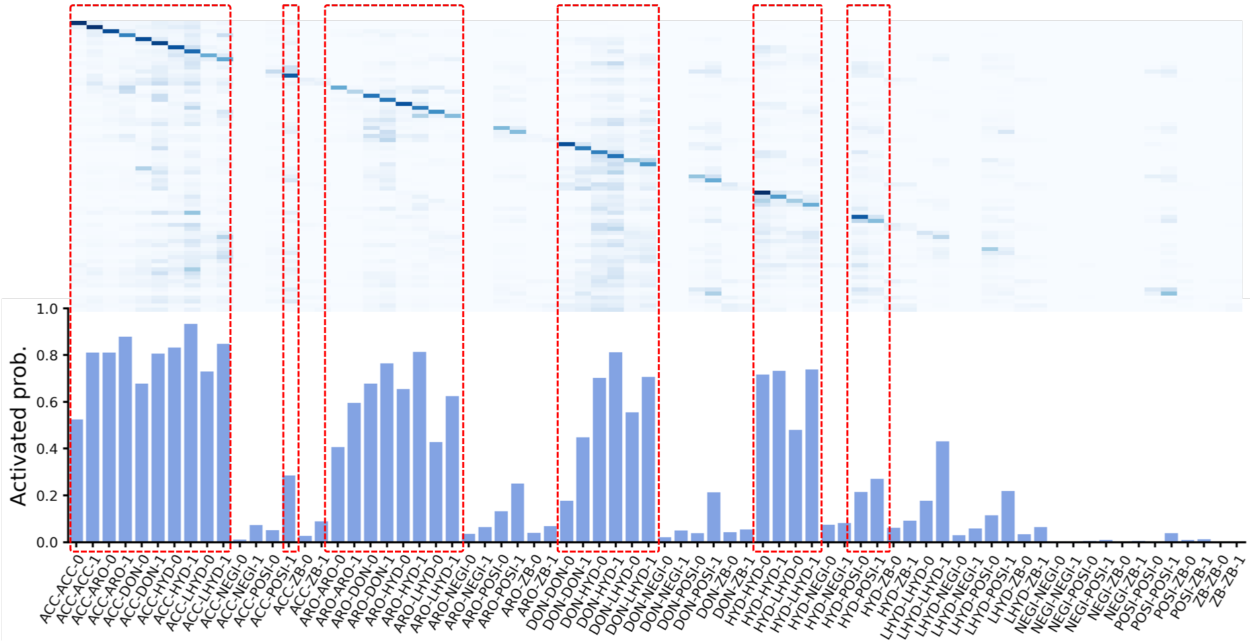

Supplementary Figure 10: Heatmap plot given one bit condition for each of 72-bit. The high-frequency bits are marked in red dash rectangles.

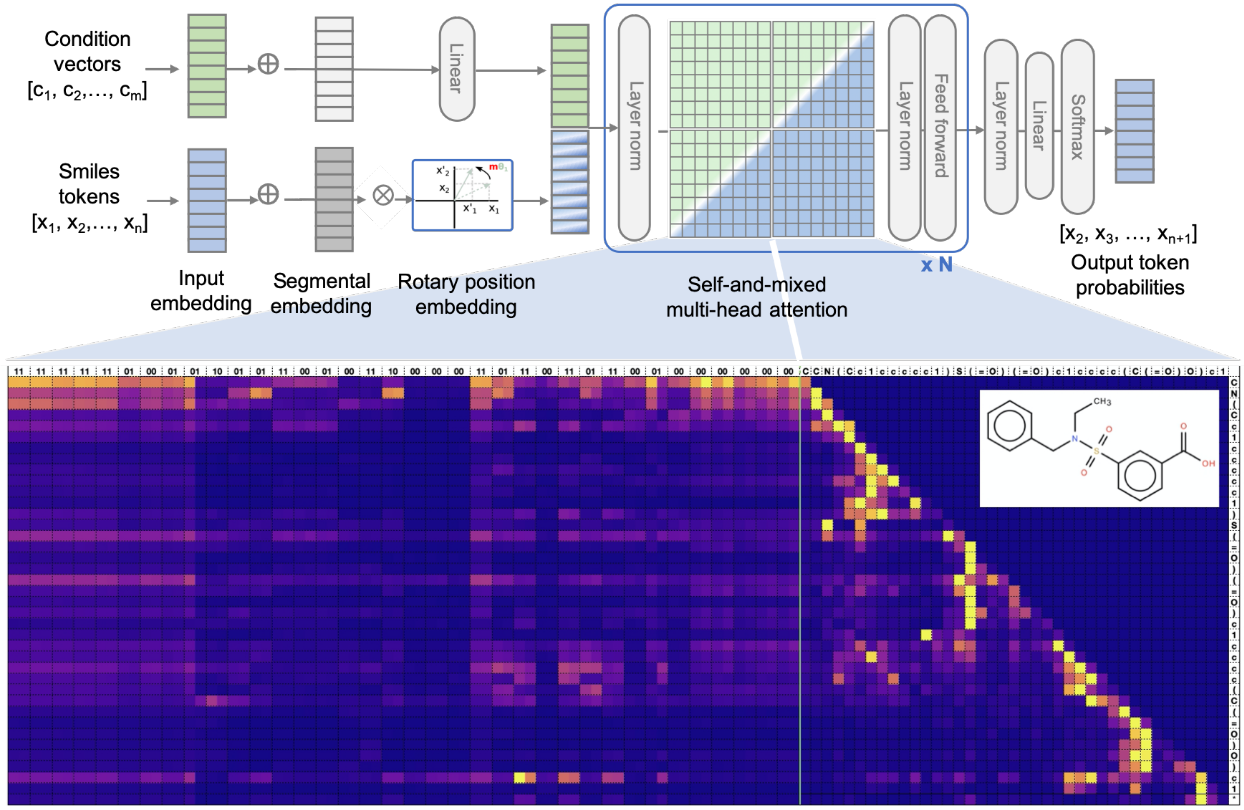

Supplementary Figure 11: Plot for model architecture and mixed attention maps between conditional vectors and SMILES tokens. For the left 72-bit subfigure, each point value is a dot product of a query embedding (a condition bit) and a key embedding (a SMILES token). For the right lower triangle, each point represents a dot product between two SMILES token embeddings. The colormap is from blue color (0) to yellow color (1).

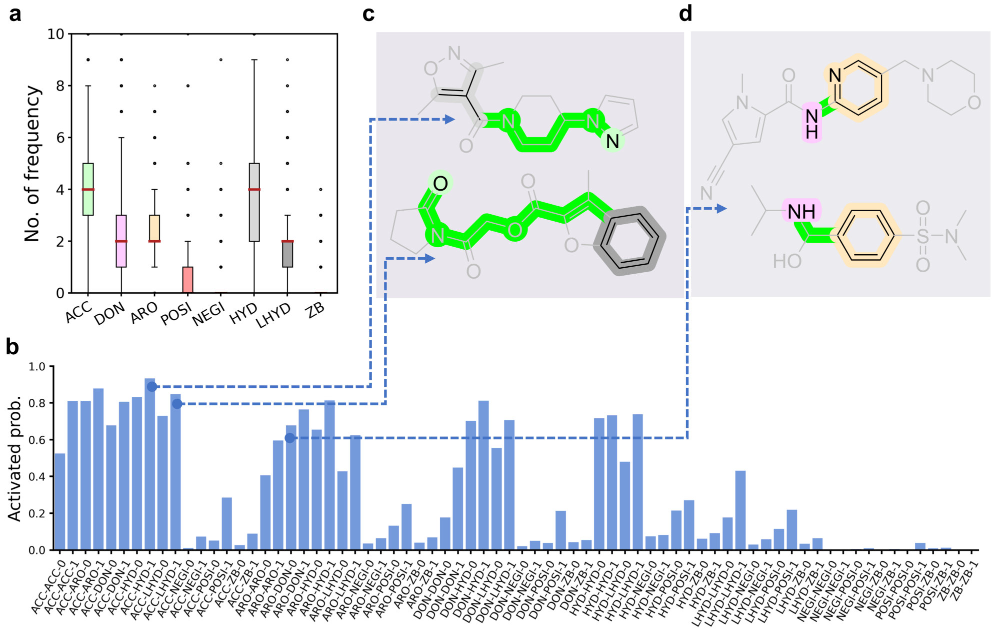

Supplementary Figure 12: Statistics on pharmacophoric features for the GuacaMol dataset. a) The averaged count plot of a molecule's pharmacophoric features. ACC: hydrogen bond acceptor; DON: hydrogen bond donor; ARO: aromatic (may play the role of hydrophobic or pi-pi stack); NEGI: negative ionizable; POSI: positive ionizable; HYD: hydrophobic; LHYD: lumped hydrophobic; ZB: Zn-ion binder. b) The averaged probability plot for each bit (of 72-bit) in a molecule. For the formula of F1-F2-0,1, F1 and F2 are potential pharmacophoric features, and the numerals 0 and 1 indicate the topological distances of (0,3) and [3,8). c-d) Bit schematic diagram for ACC-HYD-1, ACC-LHYD-1, and ARO-DON-0.

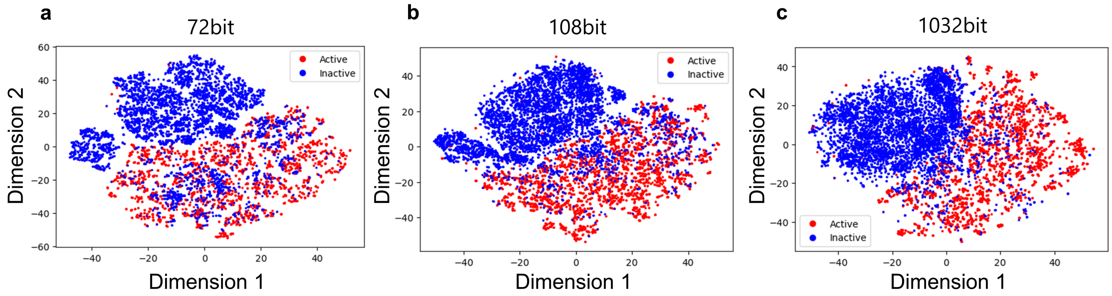

Supplementary Figure 13: T-SNE dimensional reduction plots of DRD2 actives and inactives using a) 72-bit, b) 108-bit and c) 1032-bit pharmacophore fingerprints as descriptors. DRD2 inactives used here is a random subset of original 41838 known inactive compounds from the ExCAPE-DB database[8] in order to keep the same amount as the DRD2 actives.

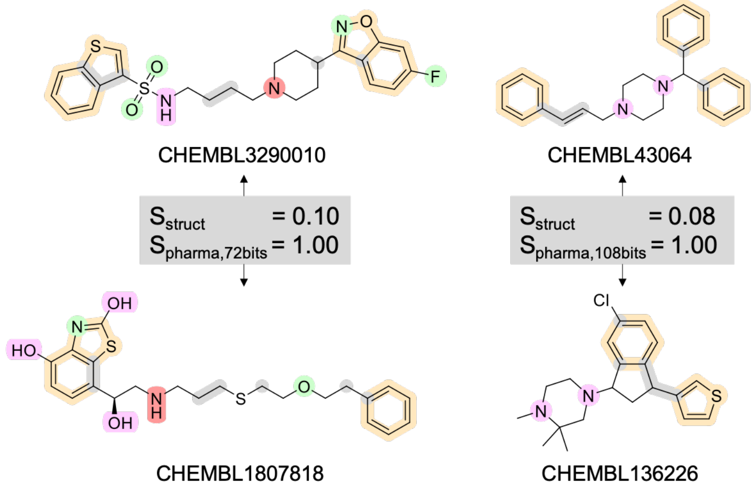

Supplementary Figure 14: Pair examples of both pharmacophorically similar and structurally dissimilar compounds (see). a) Found using 72-bit pharmacophore fingerprint. b) Found using 108-bit pharmacophore fingerprint.

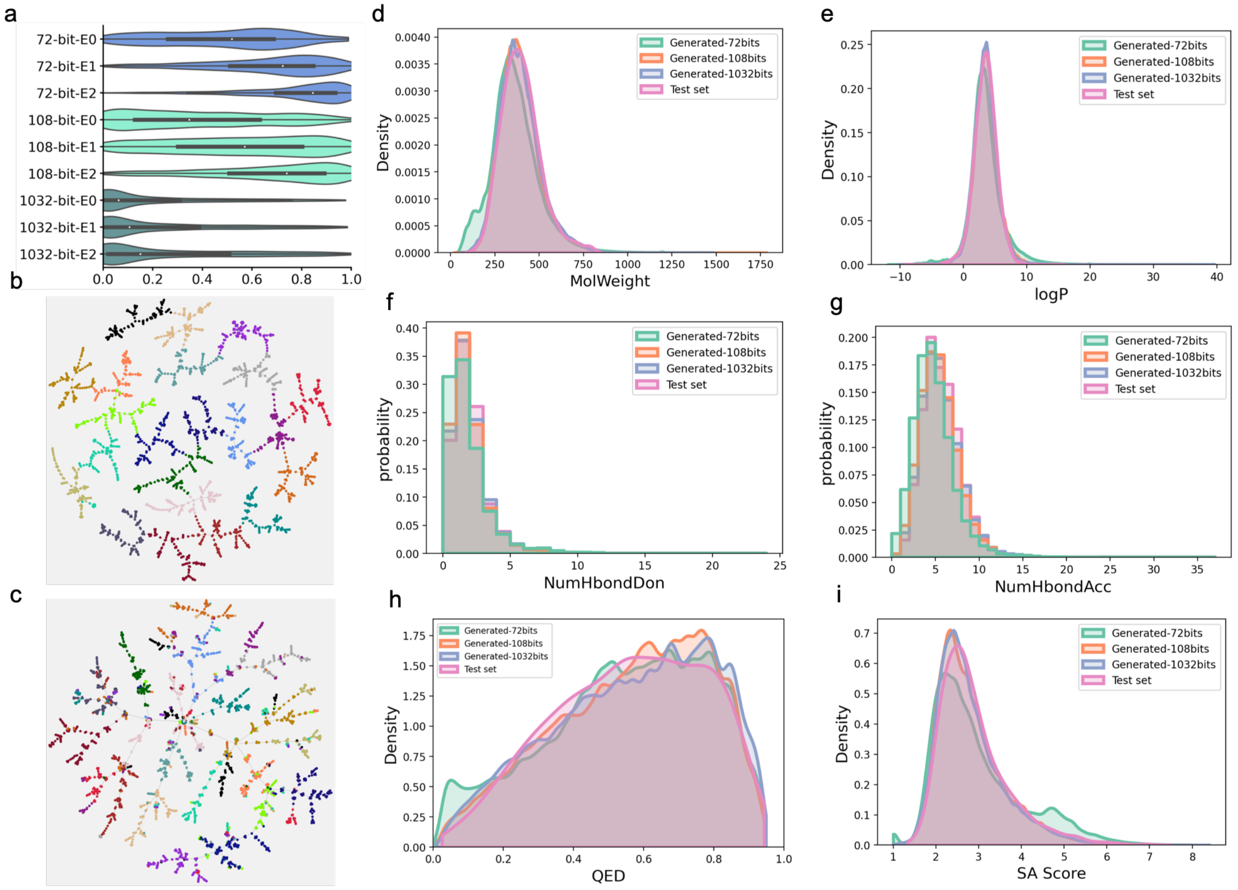

Supplementary Figure 15: Summary of model generative evaluation. a) The $E_0/E_1/E_2$ (see Section "Evaluation metrics" in main text) proportions of 1,000,000 generated molecules based on 1000 sampled condition vectors (1000 molecules per condition vector). b) and c) TMap plots based on S_{pharma} and S_{struct} for 2200 molecules generated from 22 condition vectors (100 molecules per condition vector). d)-i) Distribution plots of six chemical properties (MolWeight (MW), logP, NumHbondDon(DON), NumHbondACC (ACC), QED and SA score for the generated molecules and the test set.

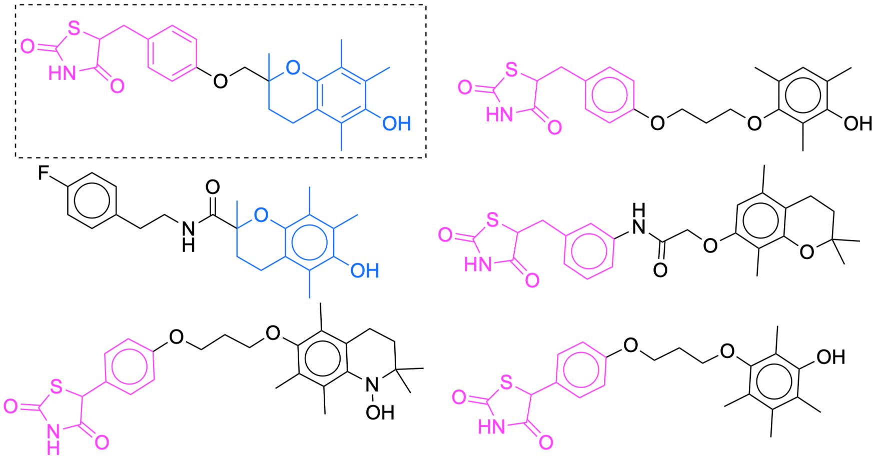

Supplementary Figure 16: 2D chemical structure diagram for Troglitazone and its similar molecules.

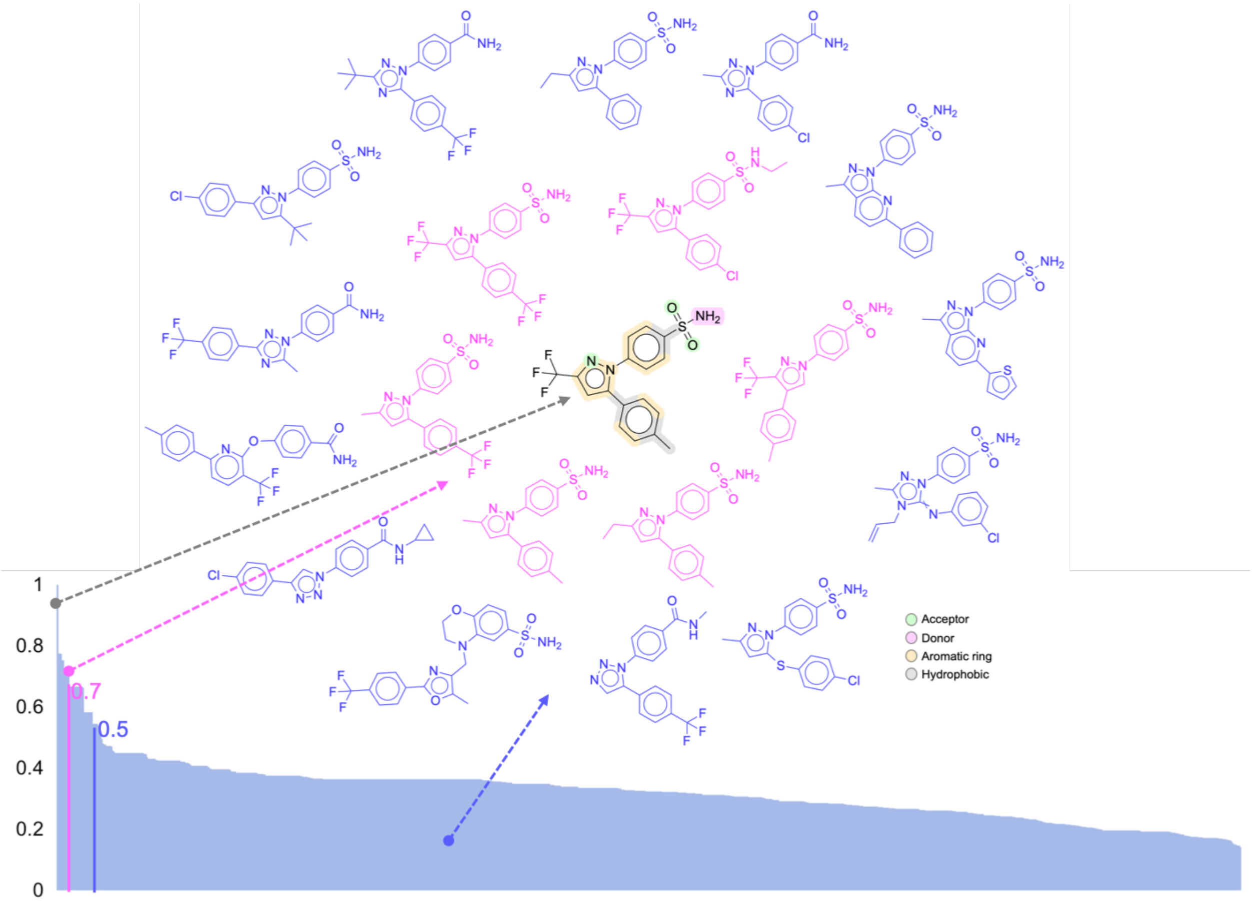

Supplementary Figure 17: Schematic diagram of the generated molecules for Celecoxib. a) Distribution plot of molecular structures based on S_{struct}. b) 2D chemical structures similar to Celecoxib sampled from different regions of S_{struct}.

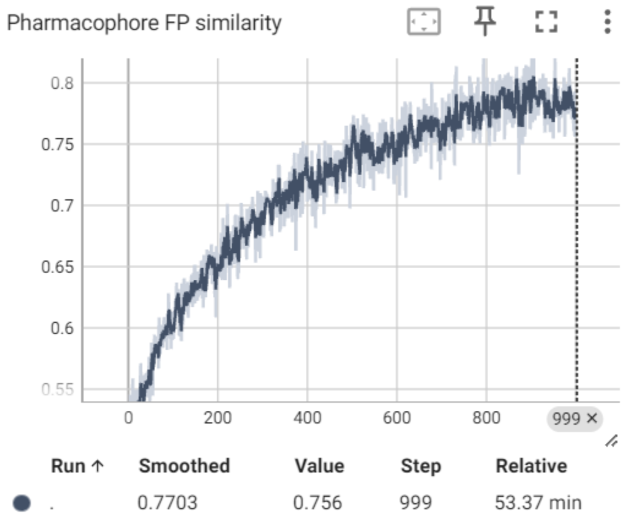

Supplementary Figure 18: The learning curve of REINVENT. The reward (y axis) reaches plateau at the end of the reinforcement learning.

Supplementary Tables

Supplementary Table 1: Supplementary results of the pharmacophore-constrained *de novo* generation task. We used the implementation of match score in the source code of PGMG (`https://github.com/CSUBioGroup/PGMG`) to evaluate generated molecules from each model.

Method	Match score
TransPharmer-72bit	0.601±0.244
TransPharmer-108bit	0.643±0.248
TransPharmer-1032bit	0.604±0.270
PGMG	0.713±0.318

Supplementary Table 2: Supplementary results of the pharmacophore-constrained *de novo* generation task. In the first column, "PGMG-max3pp" refers to PGMG using a maximum of three input features, whereas "PGMG-max8pp" denotes PGMG using a maximum of eight input features. The default PGMG evaluated on 114 test set molecules with diverse 3D conformations is labeled as "PGMG (114 cases, embed1)" for molecules adopting the first conformation and "PGMG (114 cases, embed2)" for those adopting conformations with RMSD > 2 Å. Additionally, "TransPharmer-72bit (114 cases)" indicates the evaluation of TransPharmer on the same 114 molecules. Results of other rows were obtained on the entire test set. Regarding the metrics presented in the subsequent columns, D_{MW} refers to the deviation in molecular weight of the generated molecules from the reference compound, calculated as the molecular weight of the generated molecule minus that of the reference compound. Similarly, D_{HA} represents the deviation in the number of heavy atoms.

Method	D_{count}	S_{pharma}	S_{struct}	D_{MW}	D_{HA}
LigDream	4.133±2.601	0.466±0.142	0.269±0.075	-20.429±55.272	-1.178±3.827
PGMG	9.346±3.735	0.348±0.132	0.176±0.052	7.587±89.773	1.606±6.319
PGMG-max3pp	9.725±3.738	0.301±0.112	0.168±0.043	-14.822±104.703	-0.290±7.397
PGMG-max8pp	9.610±3.708	0.362±0.125	0.176±0.049	48.468±74.591	4.762±5.106
PGMG (114 cases,embed1)	11.099±4.708	0.337±0.126	0.174±0.051	-43.501±99.374	-1.992±7.077
PGMG (114 cases,embed2)	10.717±4.392	0.342±0.132	0.176±0.051	-43.210±100.657	-1.963±7.032
TransPharmer-count	0.316±0.371	0.477±0.133	0.227±0.060	7.053±62.151	0.714±4.059
TransPharmer-72bit	4.645±2.606	0.499±0.136	0.272±0.085	11.571±78.998	0.953±5.438
TransPharmer-108bit	3.642±2.593	0.576±0.147	0.335±0.107	5.282±68.935	0.525±4.702
TransPharmer-1032bit	3.269±1.995	0.600±0.139	0.354±0.100	0.152±67.044	0.225±4.521
TransPharmer-72bit (114 cases)	5.863±2.769	0.496±0.138	0.280±0.092	-21.696±95.512	-1.352±6.643

Supplementary Table 3: Supplementary results of the pharmacophore-constrained scaffold elaboration task. In this task, each case comprises a commencing fragment and a corresponding reference final structure. We denote S_{pharma} as the mean pharmacophoric similarity, and S_{struct} as the mean topological similarity, between the commencing fragment and the reference compound. Additionally, we represent r_{MW} as the average molecular weight ratio and r_{HA} as the average ratio of heavy atoms between the commencing fragment and the reference compound. The "Small" and "Large" cases are terms used to categorize the 20 cases with the lowest and largest r_{HA} values, respectively. These cases are considered relatively complex and simple, respectively, in the context of pharmacophore-guided generation.

cases	S_{pharma}	S_{struct}	r_{MW}	r_{HA}
All	0.364±0.243	0.365±0.352	0.494±0.269	0.491±0.271
Small	0.020±0.011	0.002±0.009	0.092±0.020	0.083±0.012
Large	0.709±0.055	0.810±0.165	0.858±0.035	0.866±0.029

Supplementary Table 4: Supplementary results of the pharmacophore-constrained scaffold elaboration task. "S_{pharma} improvement" refers to the average enhancement in pharmacophoric similarity (S_{pharma}) between the generated molecules and the reference compound for each case. This improvement is compared to the similarity between the starting fragment and the reference compound. Similarly, "S_{struct} improvement" signifies the average improvement in topological similarity (S_{struct}) between the generated molecules and the reference compound. Each model's performance is assessed in all cases, small cases, and large cases, respectively.

All cases					
Method	S_{pharma}	S_{pharma} improvement	S_{struct}	S_{struct} improvement	D_{count}
DEVELOP	0.231±0.160	-0.133±0.380	0.140±0.074	-0.224±0.254	12.853±7.016
TransPharmer-count	0.706±0.218	0.333±0.216	0.521±0.221	0.149±0.091	0.190±0.248
TransPharmer-72bit	0.702±0.176	0.331±0.250	0.526±0.181	0.157±0.115	3.047±2.249
TransPharmer-108bit	0.751±0.167	0.381±0.273	0.568±0.180	0.197±0.129	2.282±1.920
TransPharmer-1032bit	0.754±0.166	0.383±0.272	0.570±0.167	0.201±0.131	2.152±1.695
Small cases					
Method	S_{pharma}	S_{pharma} improvement	S_{struct}	S_{struct} improvement	D_{count}
DEVELOP	0.215±0.112	0.213±0.114	0.117±0.047	0.097±0.042	13.096±6.742
TransPharmer-count	0.519±0.159	0.517±0.158	0.278±0.090	0.258±0.090	0.268±0.256
TransPharmer-72bit	0.556±0.136	0.554±0.136	0.319±0.084	0.299±0.081	4.457±2.118
TransPharmer-108bit	0.631±0.146	0.629±0.143	0.370±0.079	0.350±0.078	3.629±2.326
TransPharmer-1032bit	0.618±0.144	0.616±0.143	0.384±0.081	0.364±0.077	3.406±2.308
Large cases					
Method	S_{pharma}	S_{pharma} improvement	S_{struct}	S_{struct} improvement	D_{count}
DEVELOP	0.231±0.193	-0.578±0.221	0.150±0.132	-0.559±0.160	14.182±6.674
TransPharmer-count	0.932±0.070	0.123±0.127	0.811±0.081	0.102±0.091	0.128±0.206
TransPharmer-72bit	0.885±0.077	0.076±0.146	0.770±0.083	0.061±0.097	2.111±3.177
TransPharmer-108bit	0.897±0.075	0.088±0.151	0.793±0.066	0.083±0.095	1.291±0.911
TransPharmer-1032bit	0.874±0.083	0.064±0.129	0.770±0.073	0.061±0.089	1.404±1.035

Supplementary Table 5: Supplementary results of the pharmacophore-constrained *de novo* generation task and scaffold elaboration task. "Valid & Unique" represents the fraction of valid and non-duplicate molecules in the generated set.

Method	Valid & Unique	
	De Novo Generation	Scaffold Elaboration
LigDream	0.145±0.078	n.a.
PGMG	0.906±0.069	n.a.
DEVELOP	n.a.	0.607±0.331
TransPharmer-count	0.920±0.074	0.360±0.340
TransPharmer-72bit	0.810±0.156	0.341±0.302
TransPharmer-108bit	0.670±0.207	0.253±0.266
TransPharmer-1032bit	0.578±0.224	0.198±0.237

Supplementary Table 6: Results of recall rates and precision numbers of TransPharmer using active and baseline conditions under different similarity cutoffs.

Condition	#Samples	Recall (%)		
		Similarity=1.0	Similarity≥0.9	Similarity≥0.8
Active	3074684	4.95	6.18	12.10
Baseline	3298544	0.88	1.26	3.24
Condition	#Samples	Precision (count)		
		Similarity=1.0	Similarity≥0.9	Similarity≥0.8
Active	4000	2.4	4.2	15.8
Baseline	4000	0.2	0.6	2.2

Supplementary Table 7: The confidence intervals of the IC_{50} values of designed compounds and Onvansertib against PLK1.

Compound	90% CI (nM)	95% CI (nM)	99% CI (nM)
IIP0942	37.57±3.55	37.57±4.23	37.57±5.56
IIP0943	5.06±1.64	5.06±1.96	5.06±2.57
IIP0944	—	—	—
IIP0945	927.7±122.5	927.7±145.9	927.7±191.7
Onvansertib	4.80±0.65	4.80±0.77	4.80±1.01

Supplementary Table 8: SMARTS definations for Base[19] pharmacophoric features

Feature	SMARTS defination
Donor	[$([N&!H0&v3,N&!H0&+1&v4,n&H1&+0,$([$([Nv3](−C)(−C)−C)]),$([$(n[n;H1]),$(nc[n;H1])])]),$([O,S;H1;+0])]
Acceptor	[$([O;H1;v2]),$([O;H0;v2;!$(O=N−∗),$([O;−;!$(∗−N=O)]),$([o;+0])]),$([n;+0;!X3;!$([n;H1](cc)cc),$([$([N;H0]#[C&v4])]),$([N&v3;H0;$(Nc)])]),$([F;$(F−[#6]);!$(FC[F,Cl,Br,I])])]
NegIonizable	[C,S](=[O,S,P])−[O;H1,H0&−1]
PosIonizable	[$([N;H2&+0][C;!$(C=∗)]),$([N;H1&+0]([C;!$(C=∗)])[C;!$(C=∗)]),$([N;H0&+0]([C;!$(C=∗)])([C;!$(C=∗)])[C;!$(C=∗)]);!$(N[a]),$([#7;+;!$([N+]−[O−])]),$(c1ncnc1),$(NC(=N)N)]
ZnBinder	[[S;D1]−[#6],[#6]−C(=O)−C−[S;D1],[#6]−C(=O)−C−C−[S;D1],[#6]−C(=O)−N−[O;D1],[#6]−C(=O)−[O;D1],[#6]−P(=O)(−O)−[C,O,N]−[C,H]]
Aromatic	[$([a;r4,!R1&r3]1:[a;r4,!R1&r3]:[a;r4,!R1&r3]:[a;r4,!R1&r3]:1),$([a;r5,!R1&r4,!R1&r3]1:[a;r5,!R1&r4,!R1&r3]:[a;r5,!R1&r4,!R1&r3]:[a;r5,!R1&r4,!R1&r3]:[a;r5,!R1&r4,!R1&r3]:1),$([a;r6,!R1&r5,!R1&r4,!R1&r3]1:[a;r6,!R1&r5,!R1&r4,!R1&r3]:[a;r6,!R1&r5,!R1&r4,!R1&r3]:[a;r6,!R1&r5,!R1&r4,!R1&r3]:[a;r6,!R1&r5,!R1&r4,!R1&r3]:[a;r6,!R1&r5,!R1&r4,!R1&r3]:1),$([a;r7,!R1&r6,!R1&r5,!R1&r4,!R1&r3]1:[a;r7,!R1&r6,!R1&r5,!R1&r4,!R1&r3]:[a;r7,!R1&r6,!R1&r5,!R1&r4,!R1&r3]:[a;r7,!R1&r6,!R1&r5,!R1&r4,!R1&r3]:[a;r7,!R1&r6,!R1&r5,!R1&r4,!R1&r3]:[a;r7,!R1&r6,!R1&r5,!R1&r4,!R1&r3]:[a;r7,!R1&r6,!R1&r5,!R1&r4,!R1&r3]:1),$([a;r8,!R1&r7,!R1&r6,!R1&r5,!R1&r4,!R1&r3]1:[a;r8,!R1&r7,!R1&r6,!R1&r5,!R1&r4,!R1&r3]:[a;r8,!R1&r7,!R1&r6,!R1&r5,!R1&r4,!R1&r3]:[a;r8,!R1&r7,!R1&r6,!R1&r5,!R1&r4,!R1&r3]:[a;r8,!R1&r7,!R1&r6,!R1&r5,!R1&r4,!R1&r3]:[a;r8,!R1&r7,!R1&r6,!R1&r5,!R1&r4,!R1&r3]:[a;r8,!R1&r7,!R1&r6,!R1&r5,!R1&r4,!R1&r3]:[a;r8,!R1&r7,!R1&r6,!R1&r5,!R1&r4,!R1&r3]:1)]
Hydrophobe	[$([D3,D4;$([#6;+0;!$([#6;$([#6]~[#7,#8,#9])])])]),$([R0;D2;$([#6;+0;!$([#6;$([#6]~[#7,#8,#9])])])])]
LumpedHydrophobe	[$([CH;!R](−[CH3])−[CH3]),$([N;D3;+](=O)[O−]),$([C;!R](−[CH3])(−[CH3])−[CH3]),$([$([r6,!R1&r5,!R1&r4,!R1&r3]);$([R;$([c,s,S&H0&v2,Br,I,$([#6;+0;!$([#6;$([#6]~[#7,#8,#9])])])])])])]1[$([r6,!R1&r5,!R1&r4,!R1&r3]);$([R;$([c,s,S&H0&v2,Br,I,$([#6;+0;!$([#6;$([#6]~[#7,#8,#9])])])])])])][$([r6,!R1&r5,!R1&r4,!R1&r3]);$([R;$([c,s,S&H0&v2,Br,I,$([#6;+0;!$([#6;$([#6]~[#7,#8,#9])])])])])])][$([r6,!R1&r5,!R1&r4,!R1&r3]);$([R;$([c,s,S&H0&v2,Br,I,$([#6;+0;!$([#6;$([#6]~[#7,#8,#9])])])])])])][$([r6,!R1&r5,!R1&r4,!R1&r3]);$([R;$([c,s,S&H0&v2,Br,I,$([#6;+0;!$([#6;$([#6]~[#7,#8,#9])])])])])])][$([r6,!R1&r5,!R1&r4,!R1&r3]);$([R;$([c,s,S&H0&v2,Br,I,$([#6;+0;!$([#6;$([#6]~[#7,#8,#9])])])])])])]1),$([$([r5,!R1&r4,!R1&r3]);$([R;$([c,s,S&H0&v2,Br,I,$([#6;+0;!$([#6;$([#6]~[#7,#8,#9])])])])])])]1[$([r5,!R1&r4,!R1&r3]);$([R;$([c,s,S&H0&v2,Br,I,$([#6;+0;!$([#6;$([#6]~[#7,#8,#9])])])])])])][$([r5,!R1&r4,!R1&r3]);$([R;$([c,s,S&H0&v2,Br,I,$([#6;+0;!$([#6;$([#6]~[#7,#8,#9])])])])])])][$([r5,!R1&r4,!R1&r3]);$([R;$([c,s,S&H0&v2,Br,I,$([#6;+0;!$([#6;$([#6]~[#7,#8,#9])])])])])])][$([r5,!R1&r4,!R1&r3]);$([R;$([c,s,S&H0&v2,Br,I,$([#6;+0;!$([#6;$([#6]~[#7,#8,#9])])])])])])]1),$([$([r4,!R1&r3]);$([R;$([c,s,S&H0&v2,Br,I,$([#6;+0;!$([#6;$([#6]~[#7,#8,#9])])])])])])]1[$([r4,!R1&r3]);$([R;$([c,s,S&H0&v2,Br,I,$([#6;+0;!$([#6;$([#6]~[#7,#8,#9])])])])])])][$([r4,!R1&r3]);$([R;$([c,s,S&H0&v2,Br,I,$([#6;+0;!$([#6;$([#6]~[#7,#8,#9])])])])])])][$([r4,!R1&r3]);$([R;$([c,s,S&H0&v2,Br,I,$([#6;+0;!$([#6;$([#6]~[#7,#8,#9])])])])])])]1),$([r3;$([R;$([c,s,S&H0&v2,Br,I,$([#6;+0;!$([#6;$([#6]~[#7,#8,#9])])])])])])]1[r3;$([R;$([c,s,S&H0&v2,Br,I,$([#6;+0;!$([#6;$([#6]~[#7,#8,#9])])])])])])][r3;$([R;$([c,s,S&H0&v2,Br,I,$([#6;+0;!$([#6;$([#6]~[#7,#8,#9])])])])])])]1)]

Supplementary Table 9: Hyperparamters of the TransPharmer model

	Name	Value
Model Parameters	Token vocab size	94
	Number of decoder layers	8
	Number of heads	8
	Token embed dim	256
	Condition embed dim	256
	Hidden layer dim	256
	Dropout ratio	0.1
	Max sequence length	100
Training Parameters	Batch size	300
	Optimizer	adamW
	Learning rate	6E-04
	Betas	0.9,0.95
	Epochs	300
Sampling Parameters	Temperature	0.7
	Batch size	200

Supplementary Table 10: Comparison of unconditional TransPharmer with the generative models benchmarked in the GuacaMol suite in terms of distribution learning metrics.

Benchmark	AAE	Graph MCTS	SMILES LSTM	VAE	ORGAN	TransPharmer
Validity	0.822	**1.000**	0.959	0.870	0.379	0.979
Uniqueness	**1.000**	**1.000**	**1.000**	0.999	0.841	**1.000**
Novelty	**0.998**	0.994	0.912	0.974	0.686	0.956
KL Divergence	0.886	0.522	0.991	0.982	0.267	0.984
Fréchet ChemNet Distance	0.529	0.015	**0.913**	0.863	0.000	0.862
Total	4.235	3.531	4.775	4.688	2.173	**4.781**

Supplementary Table 11: Comparison of unconditional TransPharmer with the generative models benchmarked on the MOSES dataset. Bold numbers indicate that TransPharmer achieves the highest rank among the compared models for specific metrics, while underlined numbers indicate that TransPharmer ranks in the second place.

Model	Valid (↑)	Unique@1k (↑)	Unique@10k (↑)	FCD (↓)		SNN (↑)	
				Test	TestSF	Test	TestSF
Train	*1.0*	*1.0*	*1.0*	*0.008*	*0.4755*	*0.6419*	*0.5859*
HMM	0.076±0.0322	0.623±0.1224	0.5671±0.1424	24.4661±2.5251	25.4312±2.5599	0.3876±0.0107	0.3795±0.0107
NGram	0.2376±0.0025	0.974±0.0108	0.9217±0.0019	5.5069±0.1027	6.2306±0.0966	0.5209±0.001	0.4997±0.0005
Combinatorial	**1.0±0.0**	0.9983±0.0015	0.9909±0.0009	4.2375±0.037	4.5113±0.0274	0.4514±0.0003	0.4388±0.0002
CharRNN	0.9748±0.0264	**1.0±0.0**	0.9994±0.0003	**0.0732±0.0247**	**0.5204±0.0379**	0.6015±0.0206	0.5649±0.0142
AAE	0.9368±0.0341	**1.0±0.0**	0.9973±0.002	0.5555±0.2033	1.0572±0.2375	0.6081±0.0043	0.5677±0.0045
VAE	0.9767±0.0012	**1.0±0.0**	0.9984±0.0005	0.099±0.0125	0.567±0.0338	**0.6257±0.0005**	**0.5783±0.0008**
JTN-VAE	**1.0±0.0**	**1.0±0.0**	**0.9996±0.0003**	0.3954±0.0234	0.9382±0.0531	0.5477±0.0076	0.5194±0.007
LatentGAN	0.8966±0.0029	**1.0±0.0**	0.9968±0.0002	0.2968±0.0087	0.8281±0.0117	0.5371±0.0004	0.5132±0.0002
TransPharmer	0.9925±0.0005	**1.0±0.0**	0.9993±0.0002	0.3750±0.0084	0.9240±0.0153	0.6109±0.0001	0.5692±0.0002

Model	Frag (↑)		Scaf (↑)		IntDiv (↑)	IntDiv2 (↑)	Filters (↑)	Novelty (↑)
	Test	TestSF	Test	TestSF				
Train	*1.0*	*0.9986*	*0.9907*	*0.0*	*0.8567*	*0.8508*	*1.0*	*1.0*
HMM	0.5754±0.1224	0.5681±0.1218	0.2065±0.0481	0.049±0.018	0.8466±0.0403	0.8104±0.0507	0.9024±0.0489	**0.9994±0.001**
NGram	0.9846±0.0012	0.9815±0.0012	0.5302±0.0163	0.0977±0.0142	**0.8738±0.0002**	0.8644±0.0002	0.9582±0.001	0.9694±0.001
Combinatorial	0.9912±0.0004	0.9904±0.0003	0.4445±0.0056	0.0865±0.0027	0.8732±0.0002	**0.8666±0.0002**	0.9557±0.0018	0.9878±0.0008
CharRNN	**0.9998±0.0002**	0.9983±0.0003	0.9242±0.0058	**0.1101±0.0081**	0.8562±0.0005	0.8503±0.0005	0.9943±0.0034	0.8419±0.0509
AAE	0.991±0.0051	0.9905±0.0039	0.9022±0.0375	0.0789±0.009	0.8557±0.0031	0.8499±0.003	0.9960±0.0006	0.7931±0.0285
VAE	0.9994±0.0001	**0.9984±0.0003**	0.9386±0.0021	0.0588±0.0095	0.8558±0.0004	0.8498±0.0004	0.9970±0.0002	0.6949±0.0069
JTN-VAE	0.9965±0.0003	0.9947±0.0002	0.8964±0.0039	0.1009±0.0105	0.8551±0.0034	0.8493±0.0035	0.9760±0.0016	0.9143±0.0058
LatentGAN	0.9986±0.0004	0.9972±0.0007	0.8867±0.0009	0.1072±0.0098	0.8565±0.0007	0.8505±0.0006	0.9735±0.0006	0.9498±0.0006
TransPharmer	0.9971±0.0001	0.9941±0.0000	**0.9389±0.0015**	0.0922±0.0046	0.8519±0.0001	0.8461±0.0001	**0.9971±0.0002**	0.8194±0.0005

Supplementary Table 12: Comparison of TransPharmer with the generative models benchmarked in the GuacaMol suite in terms of goal directed metrics.

Benchmark	Best of Dataset	SMILES GA	Graph MCTS	Graph GA	SMILES LSTM	TransPharmer
Celecoxib rediscovery	0.505	0.732	0.355	1.000	1.000	1.000
Troglitazone rediscovery	0.419	0.515	0.311	1.000	1.000	1.000*
Thiothixene rediscovery	0456	0.598	0.311	1.000	1.000	1.000
Aripiprazole rediscovery	0.595	0.834	0.380	≥0.750	≥0.750	1.000
Albuterol rediscovery	0.719	0.907	0.749	≥0.750	≥0.750	1.000
Mestranol rediscovery	0.629	0.790	0.402	≥0.750	≥0.750	1.000

*Troglitazone is exactly rediscovered by constrained sampling starting from the 5-(4-methoxybenzyl)thiazolidine-2,4-dione moiety due to the insufficiency of the pharmacophore definition to label all methyl groups.

Supplementary Table 13: Results of comparison with REINVENT.

Model	S_{pharma}	D_{count}
REINVENT	0.41±0.12	6.1±2.2
TransPharmer-72bit	0.50±0.14	4.6±2.6

Supplementary Table 14: Results of comparison with REINVENT in the case study of PLK1.

Model	S_{pharma}	S_{pharma} (72bit)	D_{count}
REINVENT	0.491	0.805	5.848
TransPharmer-72bit	0.621	0.914	4.757

Supplementary Table 15: Results of the ablation study.

Model	*De Novo* Generation		Scaffold Elaboration	
	D_{count}	S_{pharma}	D_{count}	S_{pharma}
TransPharmer	4.6±2.6	0.50±0.14	3.0±2.2	0.70±0.18
without topo[a]	6.3±2.6	0.38±0.10	3.9±2.2	0.61±0.21
without topo and combo[b]	8.5±2.7	0.31±0.08	5.3±2.7	0.55±0.22
without any guidance[c]	10.5±2.5	0.27±0.07	6.6±3.6	0.51±0.24

[a] remove topological distance information;
[b] remove topological distance information and feature combination;
[c] no pharmacophore guidance (unconditional generation).

Supplementary Methods

Procedure for the Synthesis of IIP0942

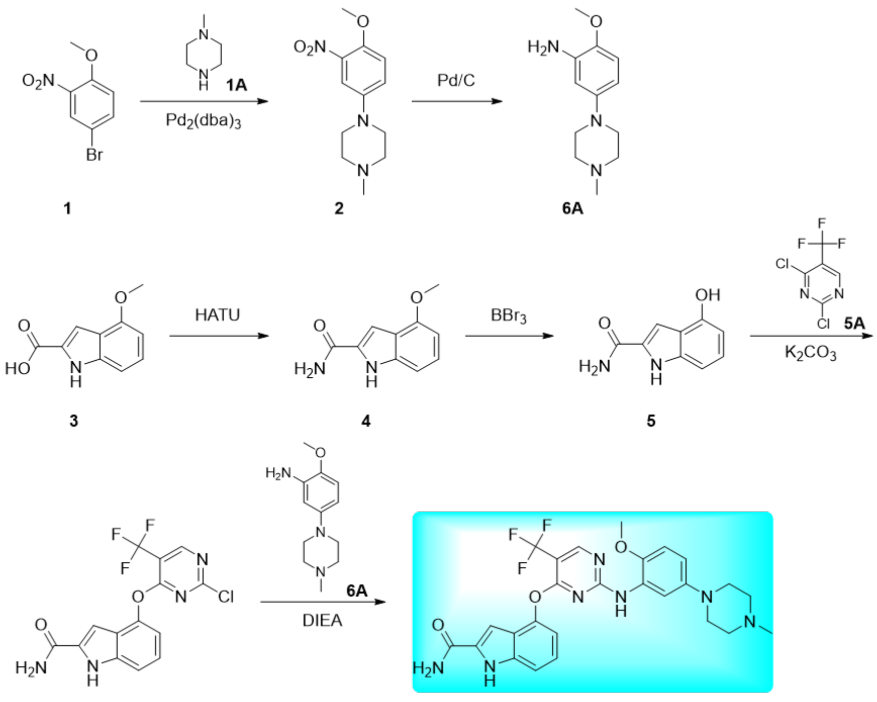

*1-(4-methoxy-3-nitrophenyl)-4-methylpiperazine (**2**)* To a solution of 4-Bromo-2-nitroanisole (5.00 g, 21.6 mmol, 1.0 eq) and 1-methylpiperazine (4.32 g, 4.78 mL, 43.1 mmol, 2.0 eq) in 1,4-Dioxane (100 mL) was added XantPhos (1.25 g, 2.15 mmol, 0.1 eq), Cs_2CO_3 (17.6 g, 4.31 mL, 53.9 mmol, 2.5 eq) and $Pd_2(dba)_3$ (987 mg, 1.08 mmol, 0.05 eq) in turn. The reaction mixture was stirred at 90 °C for 16 hours. LCMS showed major desired mass. The solution was filtered and concentrated under reduced pressure. The crude product was purified by silica gel chromatography eluted with PE:EtOAc=3:1(Rf=0.5) to afford 1-(4-methoxy-3-nitrophenyl)-4-methylpiperazine (5.00 g, Yield: 92%) as a red oil.

*2-Methoxy-5-(4-methyl-piperazin-1-yl)-phenylamine (**6A**)* To a solution of 1-(4-methoxy-3-nitrophenyl)-4-methylpiperazine (1.00 g, 3.98 mmol, 1.0 eq) in MeOH (1 mL) was added Pd/C (678 mg, 5%, 0.08 eq) under N_2. The suspension was degassed under vacuum and purged with H_2 several times. The mixture was stirred under H2 pressure at 25 °C for 16 hours. LCMS showed major desired mass. The solution was filtered and concentrated to afford 2-Methoxy-5-(4-methyl-piperazin-1-yl)-phenylamine (880 mg, Yield: 99%) as a brown solid.

*4-methoxy-1H-indole-2-carboxamide (**4**)* To a solution of 4-Methoxyindole-2-carboxylic acid (5.00 g, 26.2 mmol, 1.0 eq) in DMF (60 mL) was added DIPEA (13.5 g, 17.3 mL, 105 mmol, 4.0 eq) and HATU (11.9 g, 31.4 mmol, 1.2 eq). Cooled the reaction mixture to 0 °C, NH_4Cl (2.80 g, 1.84 mL, 52.3 mmol, 2.0 eq) was added in the mixture. The reaction mixture was stirred at 25 °C for 1 hour. LCMS showed major desired mass. The reaction mixture added EtOAc (100 mL) and H_2O (400 mL), the aqueous phase was extracted with EtOAc (80 mL) for three times. The combined organic layers were dried over Na_2SO_4, filtered and concentrated. The crude product was purified by silica gel chromatography eluted with PE: EtOAc =3:1(Rf=0.5) to afford 4-methoxy-1H-indole-2-carboxamide (3.00 g, Yield: 60%) as an off-white solid.

*4-hydroxy-1H-indole-2-carboxamide (**5**)* To a solution of 4-methoxy-1H-indole-2-carboxamide (2.90 g, 15.3 mmol, 1.0 eq) in DCM (30 mL). When cooled at 0 °C, BBr_3 (11.5 g, 47.3 mL, 45.7 mmol, 3.0 eq) was added

dropwise. The reaction mixture was stirred at 25 °C for 2 hours. LCMS showed major desired mass. The reaction mixture was quenched by methanol (50 mL) and methylammonium solution (50 mL). Then the solution was concentrated under vacuum. The crude product was purified by silica gel chromatography eluted with PE: EtOAc =20:1(Rf=0.5) to afford 4-hydroxy-1H-indole-2-carboxamide (2.00 g, Yield: 74%) as a brown solid.

*4-[2-chloro-5-(trifluoromethyl)pyrimidin-4-yl]oxy-1H-indole-2-carboxamide (**6**)* To a solution of 4-hydroxy-1H-indole-2-carboxamide (100 mg, 568 μmol, 1.0 eq) in MeCN (2 mL) was added K_2CO_3 (157 mg, 1.14 mmol, 2.0 eq). Then 2,4-Dichloro-5-(trifluoromethyl) pyrimidine (123 mg, 568 μmol, 1.0 eq) was added at 0 °C. The reaction mixture was stirred at 25 °C for 2 hours. LCMS showed major desired mass. The solution was filtered and concentrated to afford 4-[2-chloro-5-(trifluoromethyl)pyrimidin-4-yl]oxy-1H-indole-2-carboxamide (150 mg, Yield: 74%) as a red oil.

4-[(2-[2-methoxy-5-(4-methylpiperazin-1-yl)phenyl]amino-5-(trifluoromethyl)pyrimidin-4-yl)oxy]-1H-indole-2-carboxamide (IIP0942) To a solution of 2-Methoxy-5-(4-methyl-piperazin-1-yl)-phenylamine (112 mg, 505 μmol, 1.5 eq) and 4-[2-chloro-5-(trifluoromethyl)pyrimidin-4-yl]oxy-1H-indole-2-carboxamide (120 mg, 336 μmol, 1.0 eq) in 1,4-Dioxane (2.5 mL) was added TFA (115 mg, 77.3 μL, 1.01 mmol, 3.0 eq). The reaction mixture was stirred at 50 °C for 16 hours. LCMS showed major desired mass. The solution was adjusted to pH=7 8 by the addition of sat.aq Na_2CO_3 (5 mL). To the mixture added EtOAc (50 mL) and H_2O (20 mL), the aqueous phase was extracted with EtOAc (20 mL) for three times. The combined organic layers were dried over Na_2SO_4, filtered and concentrated. The residue was purified by silica gel chromatography eluted with PE: EtOAc =0:1(Rf=0.5). Then the crude product was purified by Prep-HPLC (FA condition) to afford 4-[(2-[2-methoxy-5-(4-methylpiperazin-1-yl)phenyl]amino-5-(trifluoromethyl)pyrimidin-4-yl)oxy]-1H-indole-2-carboxamide (22.07 mg, Yield: 11.75%, Purity: 97%) as a white solid.

Procedure for the Synthesis of IIP0943

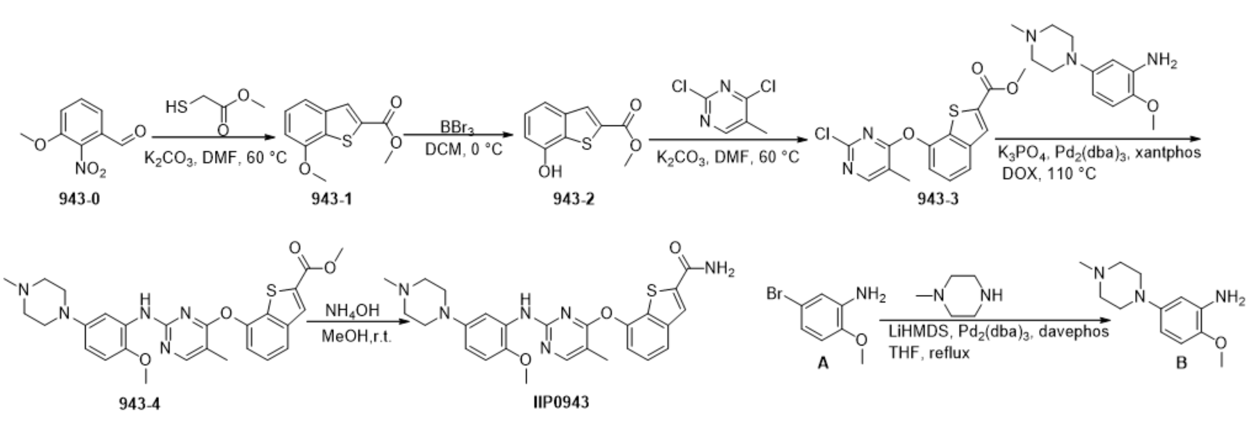

*2-methoxy-5-(4-methylpiperazin-1-yl)aniline (**B**)* To a solution of 5-bromo-2-methoxyaniline (A, 2.0 g, 9.90 mmol), 1-methylpiperazine (1.5 g, 14.85 mmol), Pd_2dba_3 (906 mg, 0.99 mmol) and davephos (778 mg, 1.98 mmol) in THF (20 mL) was added LiHMDS (1.0 N in hexane, 19.8 mL, 19.80 mmol) at room temperature. The reaction mixture was stirred at 70 °C for 16 hours. After LCMS indicated the reaction is completed. The mixture was extracted with EtOAc (30 mLx3). The combined organic layer was washed by brine, dried over Na_2SO_4, filtered and concentrated at 45 °C under reduced pressure. The residue was purified by flash column chromatography (20 g, DCM/ MeOH = 100:00 93:7) to give 2-methoxy-5-(4-methylpiperazin-1-yl)aniline (**B**, 1.5 g, 90.47% purity, 69% yield) as a yellow solid.

*methyl 7-methoxybenzo[b]thiophene-2-carboxylate (**943-1**)* A mixture of 3-methoxy-2-nitrobenzaldehyde (**943-0**, 1.2 g, 6.76 mmol) and methyl 2-mercaptoacetate (788 mg, 7.43 mmol) and K_2CO_3 (1.9 g, 13.51 mmol) in DMF (20 mL) was stirred at 60 °C for 16 hours. After LCMS indicated the reaction completed, the

reaction mixture was quenched with water (20 mL), extracted with ethyl acetate (30 mLx3). The combined organic layer was washed by saturated NH_4Cl (30 mL), dried over Na_2SO_4, filtered and concentrated at 45 °C under reduced pressure. The residue was purified by flash column chromatography (20 g cartridge, 30 100% petroleum ether/ethyl acetate, eluting at 70%) to give methyl 7-methoxybenzo[b]thiophene-2-carboxylate (**943-1**, 1.0 g, 100% purity, 68% yield) as a yellow solid.

*methyl 7-hydroxybenzo[b]thiophene-2-carboxylate (**943-2**)* To a solution of methyl 7-methoxybenzo[b]thiophene-2-carboxylate (**943-1**, 1.0 g, 4.50 mmol) in DCM (10 mL) was added BBr_3 (1.7 g, 6.76 mmol) stirred at 0 °C for 1 hour. After LCMS indicated the reaction completed, the reaction mixture was quenched with water (10 mL), extracted with DCM (20 mLx2). The combined organic layer was washed by saturated NaCl (20 mL), dried over Na_2SO_4, filtered and concentrated at 45 °C under reduced pressure. The residue was purified by flash column chromatography (20 g cartridge, 50 100% petroleum ether/ethyl acetate, eluting at 85%) to give methyl 7-hydroxybenzo[b]thiophene-2-carboxylate (**943-2**, 667 mg, 54.06% purity, 71% yield) as a yellow solid.

*methyl 7-((2-chloro-5-methylpyrimidin-4-yl)oxy)benzo[b]thiophene-2-carboxylate (**943-3**)* A mixture of methyl 7-hydroxybenzo[b]thiophene-2-carboxylate (**943-2**, 240 mg, 1.15 mmol) and 2,4-dichloro-5-methylpyrimidine (188 mg, 1.15 mmol) and K_2CO_3 (319 mg, 2.31 mmol) in DMF (5 mL) was stirred at 60 °C for 16 hours. After LCMS indicated the reaction completed, the reaction mixture was quenched with water (10 mL), extracted with ethyl acetate (20 mLx3). The combined organic layer was washed by saturated NH_4Cl (20 mL), dried over Na_2SO_4, filtered and concentrated at 45 °C under reduced pressure. The residue was purified by flash column chromatography (12 g cartridge, 40 100% petroleum ether/ethyl acetate, eluting at 55%) to give methyl 7-((2-chloro-5-methylpyrimidin-4-yl)oxy)benzo[b]thiophene-2-carboxylate (**943-3**, 240 mg, 90.96% purity, 62% yield) as a yellow solid.

*methyl 7-((2-((2-methoxy-5-(4-methylpiperazin-1-yl)phenyl)amino)-5-methylpyrimidin-4-yl)oxy)benzo[b]thiophene-2-carboxylate (**943-4**)* To a solution of methyl 7-((2-chloro-5-methylpyrimidin-4-yl)oxy)benzo[b]thiophene-2-carboxylate (**943-3**, 240 mg, 0.72 mmol), 2-methoxy-5-(4-methylpiperazin-1-yl)aniline (**B**, 159 g, 0.72 mmol), Pd_2dba_3 (66 mg, 0.07 mmol) and Xantphos (83 mg, 0.14 mmol) in DOX (3 mL) was added K_3PO_4 (457 mg, 2.16 mmol) at room temperature. The reaction mixture was stirred at 110 °C for 16 hours. After LCMS indicated the reaction is completed, The mixture was extracted with EtOAc (10 mLx3). The combined organic layer was washed by brine, dried over Na_2SO_4, filtered and concentrated at 45 °C under reduced pressure. The residue was purified by flash column chromatography (12 g cartridge, 30 100% petroleum ether/ethyl acetate, eluting at 60%) to give methyl 7-((2-((2-methoxy-5-(4-methylpiperazin-1-yl)phenyl)amino)-5-methylpyrimidin-4-yl)oxy) benzo[b]thiophene-2-carboxylate (**943-4**, 200 mg, 66.82% purity, 54% yield) as a yellow solid.

7-((2-((2-methoxy-5-(4-methylpiperazin-1-yl)phenyl)amino)-5-methylpyrimidin-4-yl)oxy) benzo[b]thiophene-2-carboxamide (IIP0943) A solution of methyl 7-((2-((2-methoxy-5-(4-methylpiperazin-1-yl)phenyl)amino)-5-methylpyrimidin-4-yl)oxy)benzo[b]thiophene-2-carboxylate (**943-4**, 200 mg, 0.39 mmol), NH_3 ((in MeOH, 7.0 mmol/mL, 1.1 mL, 7.80 mmol) in MeOH (2 mL) was stirred at room temperature for 16 hours. After completion of the reaction indicated by LCMS, the reaction mixture was concentrated at 45 °C under reduced pressure. The residue was which was purified by prep-HPLC to give 2 7-((2-((2-methoxy-5-(4-methylpiperazin-1-yl)phenyl)amino)-5-methylpyrimidin-4-yl)oxy)benzo[b]thiophene-2-carboxamide (IIP0943, 54.87 mg, 98.59% purity, 28% yield) as a white solid.

Procedure for the Synthesis of IIP0944

*5-(2-iodophenyl)-3-methyl-1,2,4-oxadiazole (**944-1**)* To a solution of N-hydroxyacetimidamide (565 mg, 7.63 mmol) in EtOH (10 mL) was added EtONa (in EtOH, 2.5 mmol/mL, 3.1 mL, 7.63 mmol) and stirred at room temperature for 0.5 h. After the reaction was added methyl 2-iodobenzoate (**944-0**, 1.0 g, 3.81 mmol), and the reaction mixture was stirred at 80 °C overnight. After LCMS indicated the reaction completed, the reaction mixture was concentrated at 45 °C under reduced pressure. The residue was purified by flash column chromatography (12 g cartridge, 0 100% petroleum ether/ethyl acetate, eluting at 30%) to give 5-(2-iodophenyl)-3-methyl-1,2,4-oxadiazole (**944-1**, 500 mg, 93.17% purity, 46% yield) as a yellow solid.

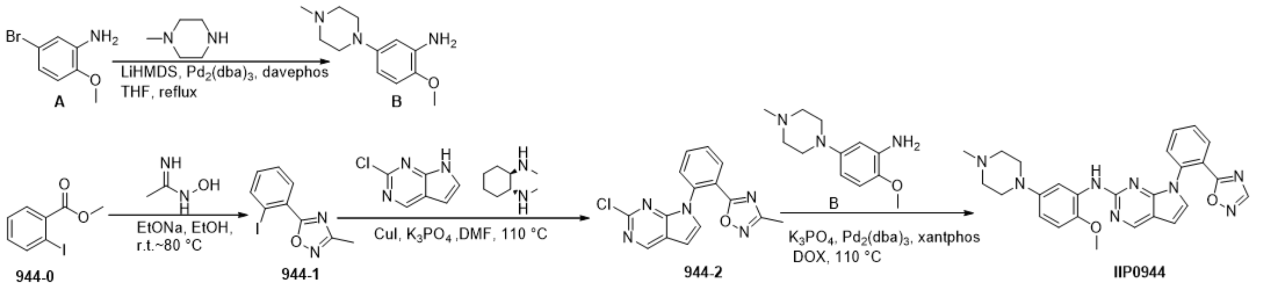

*5-(2-(2-chloro-7H-pyrrolo[2,3-d]pyrimidin-7-yl)phenyl)-3-methyl-1,2,4-oxadiazole (**944-2**)* A mixture of sodium 5-(2-iodophenyl)-3-methyl-1,2,4-oxadiazole(**944-1**, 500 mg, 1.74 mmol), 2-chloro-7H-pyrrolo[2,3-d]pyrimidine0 (267 mg, 1.75 mmol), (1R,2R)-N1,N2-dimethylcyclohexane-1,2-diamine (74 mg, 0.52 mmol), K_3PO_4 (1.1 g, 5.24 mmol) and CuI (66 mg, 0.35 mmol) in DMF(10 mL) under N_2 protection was stirred at 110 °C overnight. After LCMS indicated the reaction is completed, the reaction mixture was cooled to room temperature and filtered. The filtered cake was washed with DCM and the filtrate was concentrated and purified by flash column chromatography (12 g, PE/EA = 100:0 63:35) to give 5-(2-(2-chloro-7H-pyrrolo[2,3-d]pyrimidin-7-yl)phenyl)-3-methyl-1,2,4-oxadiazole (**944-2**, 180 mg, 100% purity, 33% yield) as yellow oil.

N-(2-methoxy-5-(4-methylpiperazin-1-yl)phenyl)-7-(2-(3-methyl-1,2,4-oxadiazol-5-yl)phenyl)-7H-pyrrolo[2,3-d]pyrimidin-2-amine (IIP0944) To a solution of 5-(2-(2-chloro-7H-pyrrolo[2,3-d]pyrimidin-7-yl)phenyl)-3-methyl-1,2,4-oxadiazole (**944-2**, 180 mg, 0.58 mmol), 2-methoxy-5-(4-methylpiperazin-1-yl)aniline (**B**, 128 mg, 0.58 mmol), Brettphos-G3-Pd (105 mg, 0.12 mmol) in toluene (3 mL) was added Cs_2CO_3(377 mg, 1.16 mmol) at room temperature. After the reaction mixture was degassed by N_2, it was stirred at 115 °C 2h under microwave. After LCMS indicated the reaction is completed, The mixture was extracted with EtOAc (10 mLx3). The combined organic layer was washed by brine, dried over Na_2SO_4, filtered and concentrated at 45 °C under reduced pressure. The residue was purified by flash column chromatography (12 g, PE/EA = 100:0 65:35) to give N-(2-methoxy-5-(4-methylpiperazin-1-yl)phenyl)-7-(2-(3-methyl-1,2,4-oxadiazol-5-yl)phenyl)-7H-pyrrolo[2,3-d]pyrimidin-2-amine (IIP0944, 6.7 mg, 98.46% purity, 2% yield) as white solid.

Procedure for the Synthesis of IIP0945

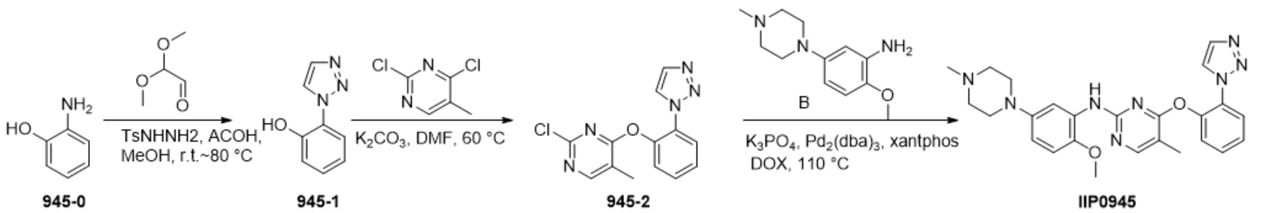

*2-(1H-1,2,3-triazol-1-yl)phenol (**945-1**)* To a solution of 2,2-dimethoxyacetaldehyde (2.1 g, 20.64 mmol) and $TsNHNH_2$ (3.8 g, 19.8 mL, 20.64 mmol) in MeOH (80 mL) stirred at room temperature for 1 hour. Then the mixture was added A_COH (825 mg, 13.76 mmol) and 2-aminophenol (**945-0**, 1.5 g, 13.76 mmol) at 80 °C for 16 hours. After LCMS indicated the reaction is completed. The mixture filtered and concentrated at 45 °C under reduced pressure. The residue was purified by flash column chromatography (20 g, DCM/ MeOH = 100:00 93:7) to give 2-(1H-1,2,3-triazol-1-yl)phenol (**945-1**, 1.1 g, 54.21% purity, 50% yield) as a yellow solid.

*4-(2-(1H-1,2,3-triazol-1-yl)phenoxy)-2-chloro-5-methylpyrimidine (**945-2**)* A mixture of methyl 2-(1H-1,2,3-triazol-1-yl)phenol (**945-1**, 1.1 g, 6.83 mmol), 2,4-dichloro-5-methylpyrimidine (1.1 g, 6.83 mmol) and

K_2CO_3 (1.9 g, 13.66 mmol) in DMF (15 mL) was stirred at 60 °C for 16 hours. After LCMS indicated the reaction completed, the reaction mixture was quenched with water (15 mL), extracted with ethyl acetate (30 mLx3). The combined organic layer was washed by saturated NH_4Cl (30 mL), dried over Na_2SO_4, filtered and concentrated at 45 °C under reduced pressure. The residue was purified by flash column chromatography (20 g cartridge, 80 100% petroleum ether/ethyl acetate, eluting at 55%) to give 4-(2-(1H-1,2,3-triazol-1-yl)phenoxy)-2-chloro-5-methylpyrimidine (**945-2**, 1.2 g, 48.92% purity, 61% yield) as a yellow solid.

4-(2-(1H-1,2,3-triazol-1-yl)phenoxy)-N-(2-methoxy-5-(4-methylpiperazin-1-yl)phenyl)-5-methylpyrimidin-2-amine (IIP0945) To a solution of methyl 4-(2-(1H-1,2,3-triazol-1-yl)phenoxy)-2-chloro-5-methylpyrimidine (**945-2**, 207 mg, 0.72 mmol), 2-methoxy-5-(4-methylpiperazin-1-yl)aniline (**B**, 159 mg, 0.72 mmol), Pd_2dba_3 (66 mg, 0.07 mmol) and Xantphos (83 mg, 0.14 mmol) in DOX (3 mL) was added K_3PO_4 (457 mg, 2.16 mmol) at room temperature. The reaction mixture was stirred at 110 °C for 16 hours under nitrogen atmosphere. After LCMS indicated the reaction is completed, The mixture was extracted with EtOAc (10 mLx3). The combined organic layer was washed by brine, dried over Na_2SO_4, filtered and concentrated at 45 °C under reduced pressure. The residue was purified by prep-HPLC to give 4-(2-(1H-1,2,3-triazol-1-yl)phenoxy)-N-(2-methoxy-5-(4-methylpiperazin-1-yl)phenyl)-5-methylpyrimidin-2-amine (IIP0945, 53.11 mg, 98.72% purity, 16% yield) as a white solid.

HRMS, HPLC, ^{1}H and ^{13}C NMR spectra of synthetic compounds

IIP0942

^{1}H NMR (400 MHz, DMSO-d_6) δ 11.80 (s, 1 H), 8.66 (s, 2 H), 8.17 (s, 1 H), 8.0 (s, 1 H), 7.34 (d, J = 8.0 Hz, 2 H), 7.20 (t, J = 8.0 Hz, 1 H), 7.03 (s, 1 H), 6.95 – 6.91 (m, 2 H), 6.83 (d, J = 8.0 Hz, 2 H), 6.59 (s, 1 H), 3.66 (s, 3 H), 2.83 – 2.67 (m, 4 H), 2.43 – 2.35 (m, 4 H), 2.21 (s, 3 H).

^{13}C NMR (101 MHz, DMSO-d_6) δ 166.51, 164.08, 163.89, 162.82, 162.24, 158.30, 158.25, 145.51, 145.29, 138.77, 132.72, 128.34, 127.41, 125.66, 124.03, 122.98, 120.93, 113.32, 112.35, 111.88, 110.83, 101.28, 100.95, 100.62, 100.03, 56.36, 54.81, 54.71, 49.09, 45.77, 45.62.

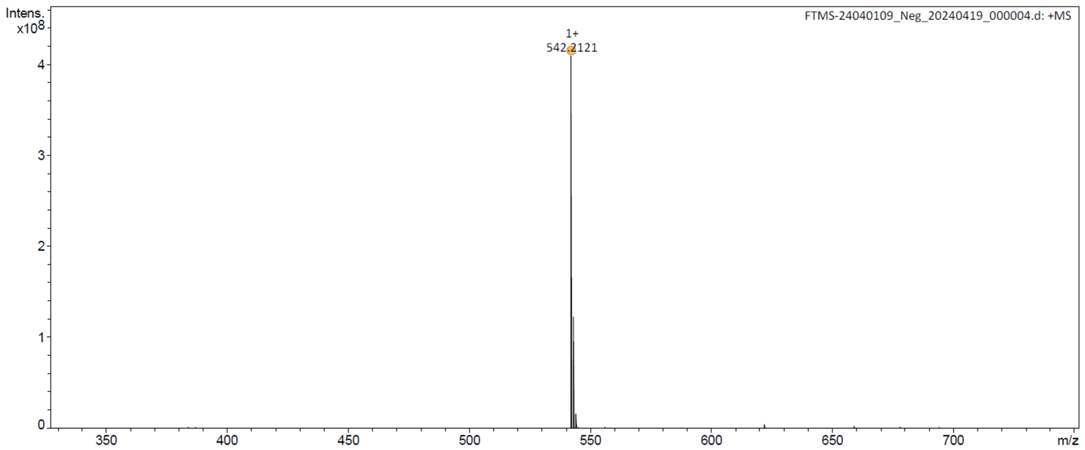

Meas. m/z	#	Ion Formula	Score	m/z	err [ppm]	Mean err [ppm]	mSigma	rdb	e⁻ Conf	N-Rule
542.2121	1	C26H27F3N7O3	100.00	542.2122	0.2	7.3	13.7	16.0	even	ok

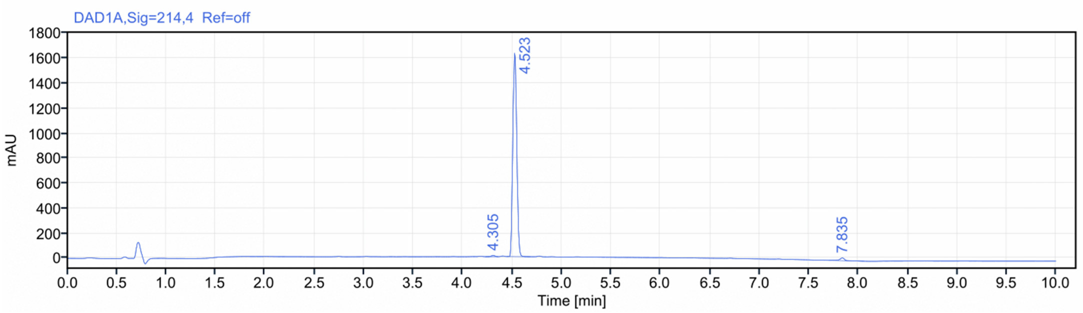

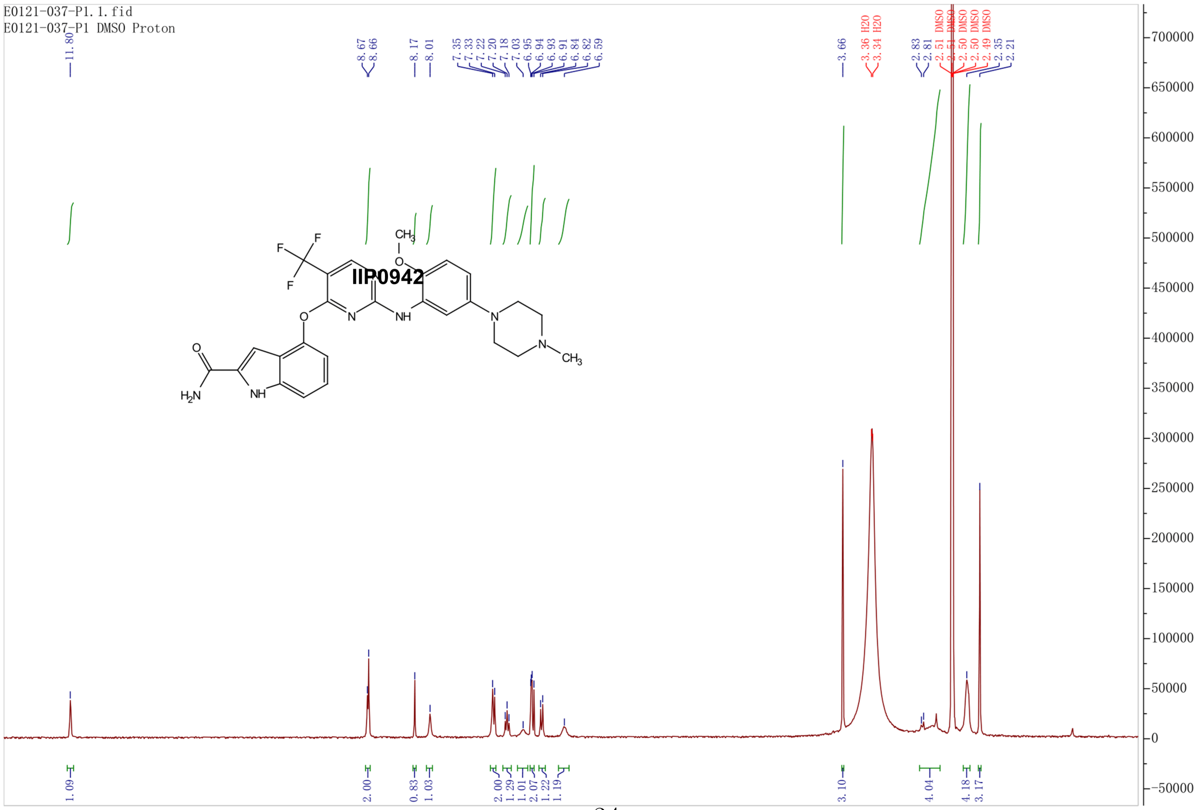

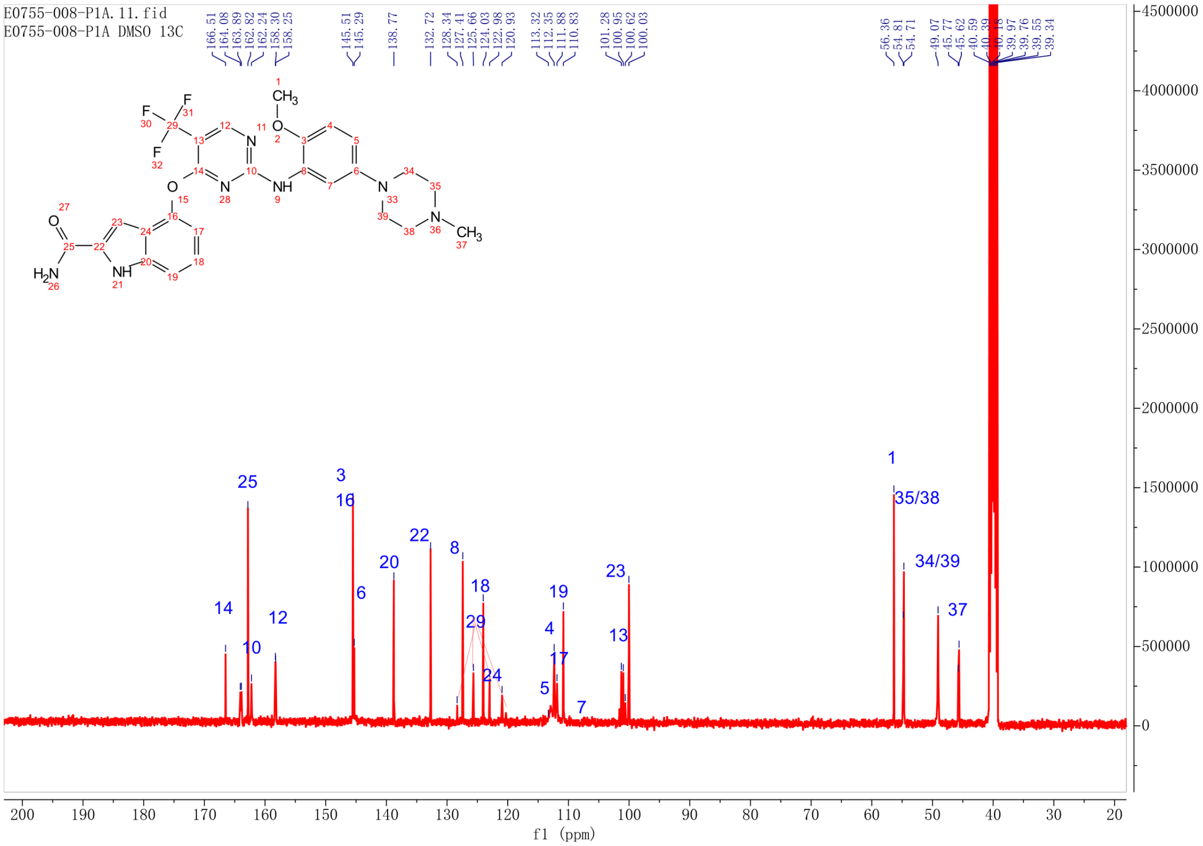
E0755-008-P1A.11.fid
E0755-008-P1A DMSO 13C
166.51
164.08
163.89
162.82
162.24
158.30
158.25
145.51
145.29
138.77
132.72
128.34
127.41
125.66
124.03
122.98
120.93
113.32
112.35
111.88
110.83
101.28
100.95
100.62
100.03
56.36
54.81
54.71
49.07
45.77
45.62
40.59
40.39
40.18
39.97
39.76
39.55
39.34
f1 (ppm)

IIP0943

^{1}H NMR (400 MHz, DMSO-d_6) 8.34 (s, 1 H), 8.26 (s, 1 H), 8.14 (s, 1 H), 7.83 (dd, J = 8.0, 0.8 Hz, 1 H), 7.66 (d, J = 6.4 Hz, 2 H), 7.49 (t, J = 7.6 Hz, 1 H), 7.41-7.39 (m, 2 H), 6.80 (d, J = 8.8 Hz, 1 H), 6.45-6.42 (m, 1 H), 3.70 (s, 3 H), 2.66 (t, J = 4.8 Hz, 4 H), 2.31 (t, J = 4.8 Hz, 4 H), 2.24 (s, 3 H), 2.21 (s, 3 H).

^{13}C NMR (151 MHz, CD_3SOCD_3) δ 166.62, 163.41, 160.33, 158.58, 147.67, 145.75, 142.76, 142.10, 141.49, 132.96, 129.07, 126.68, 126.12, 122.69, 118.65, 111.64, 110.04, 108.71, 108.50, 56.45, 55.19, 49.58, 46.27, 12.24.

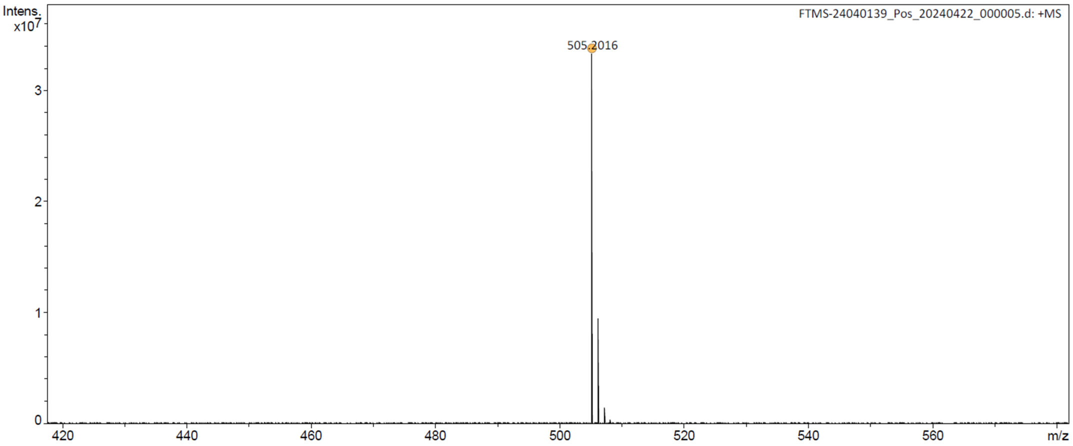

Meas. m/z	#	Ion Formula	Score	m/z	err [ppm]	Mean err [ppm]	mSigma	rdb	e⁻ Conf	N-Rule
505.2016	1	C26H29N6O3S	100.00	505.2016	0.0	-0.3	18.9	18.0	even	ok

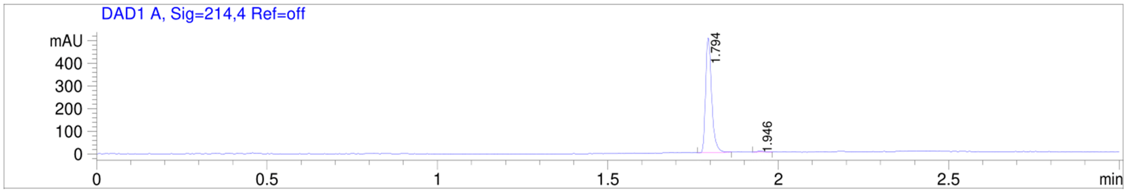

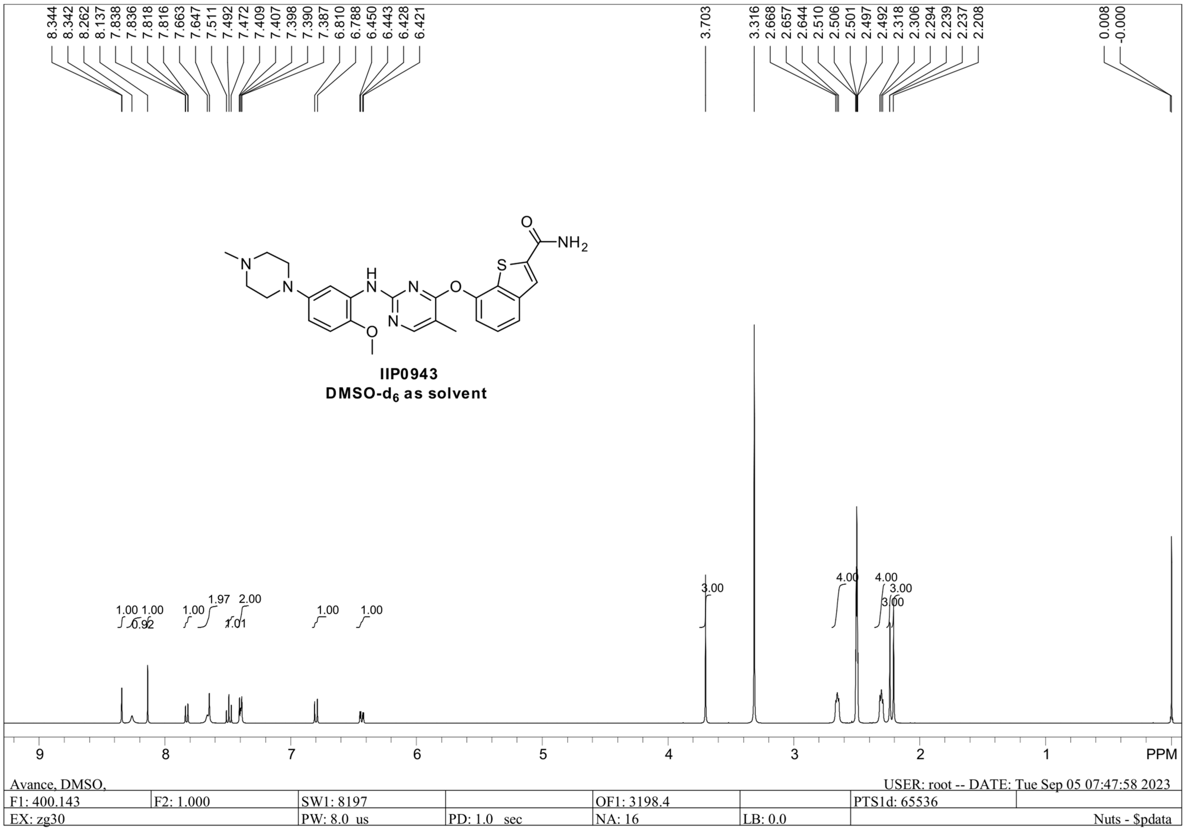

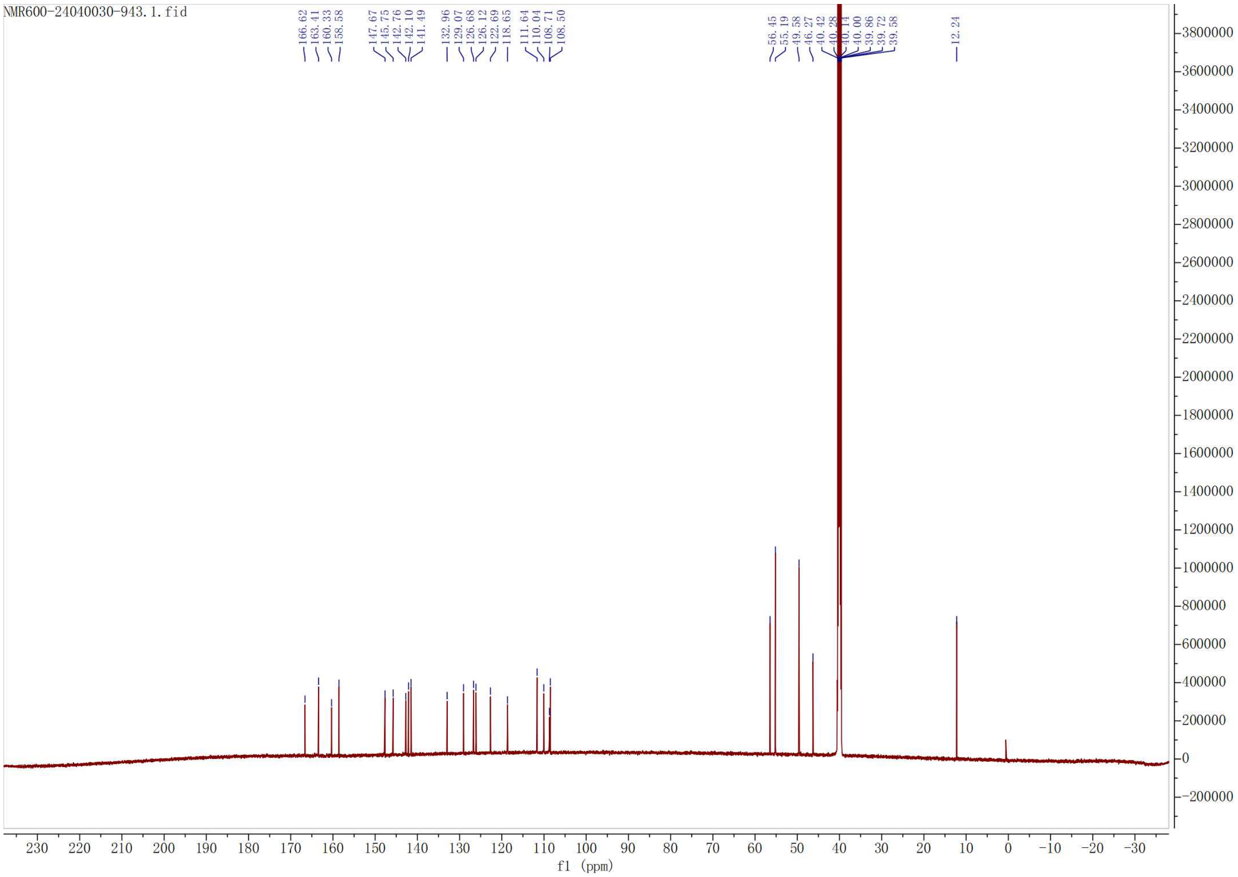
NMR600-24040030-943.1.fid
166.62
163.41
160.33
158.58
147.67
145.75
142.76
142.10
141.49
132.96
129.07
126.68
126.12
122.69
118.65
111.64
110.04
108.71
108.50
56.45
55.19
49.58
46.27
40.42
40.28
40.14
40.00
39.86
39.72
39.58
12.24
f1 (ppm)

IIP0944

^{1}H NMR (400 MHz, DMSO-d_6) δ 8.78 (s, 1 H), 8.22 (d, J = 6.8 Hz, 1 H), 7.91 (t, J = 7.6 Hz, 1 H), 7.81-7.73 (m, 3 H), 7.64 (s, 1 H), 7.41 (d, J = 3.6 Hz, 1 H), 6.82 (d, J = 8.8 Hz, 1 H), 6.69 (d, J = 3.6 Hz, 1 H), 6.39 (dd, J = 8.8, 2.8 Hz, 1 H), 3.78 (s, 3 H), 2.73 (d, J = 4.8 Hz, 4 H), 2.35 (t, J = 4.0 Hz, 4 H), 2.25 (s, 3 H), 2.14 (s, 3 H).

^{13}C NMR (151 MHz, CD_3SOCD_3) δ 173.82, 167.43, 156.05, 152.71, 151.72, 145.90, 141.42, 136.14, 134.25, 131.31, 129.99, 129.84, 129.67, 128.38, 122.13, 112.95, 111.27, 108.42, 106.63, 102.17, 56.65, 55.34, 49.50, 46.34, 11.47.

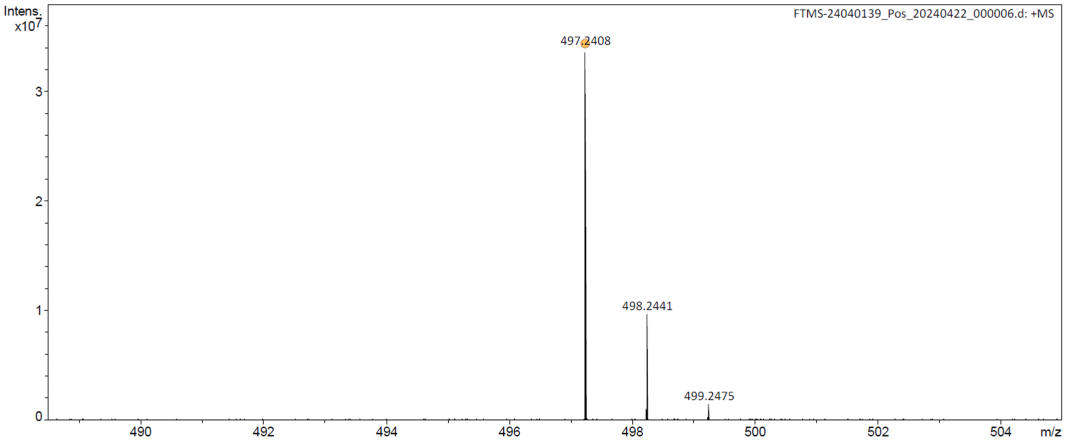

Meas. m/z	#	Ion Formula	Score	m/z	err [ppm]	Mean err [ppm]	mSigma	rdb	e⁻ Conf	N-Rule
497.2408	1	C27H29N8O2	100.00	497.2408	0.0	-0.5	23.4	18.0	even	ok

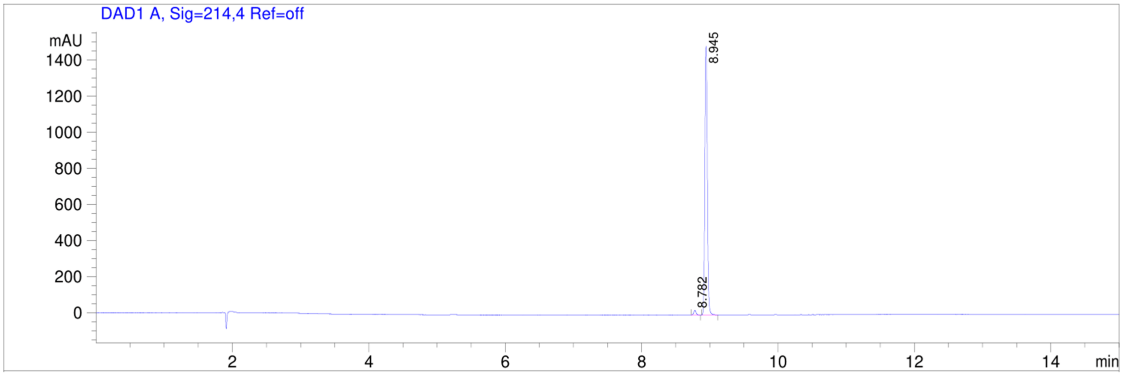

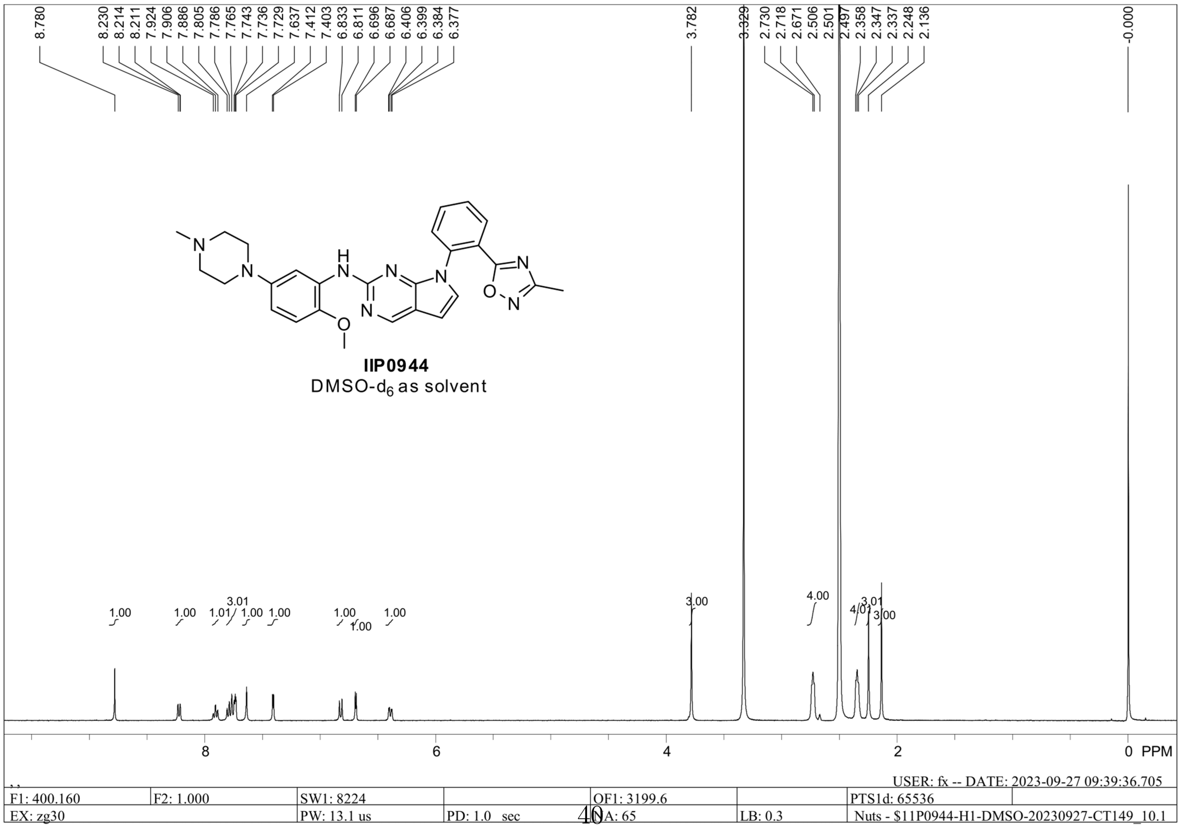

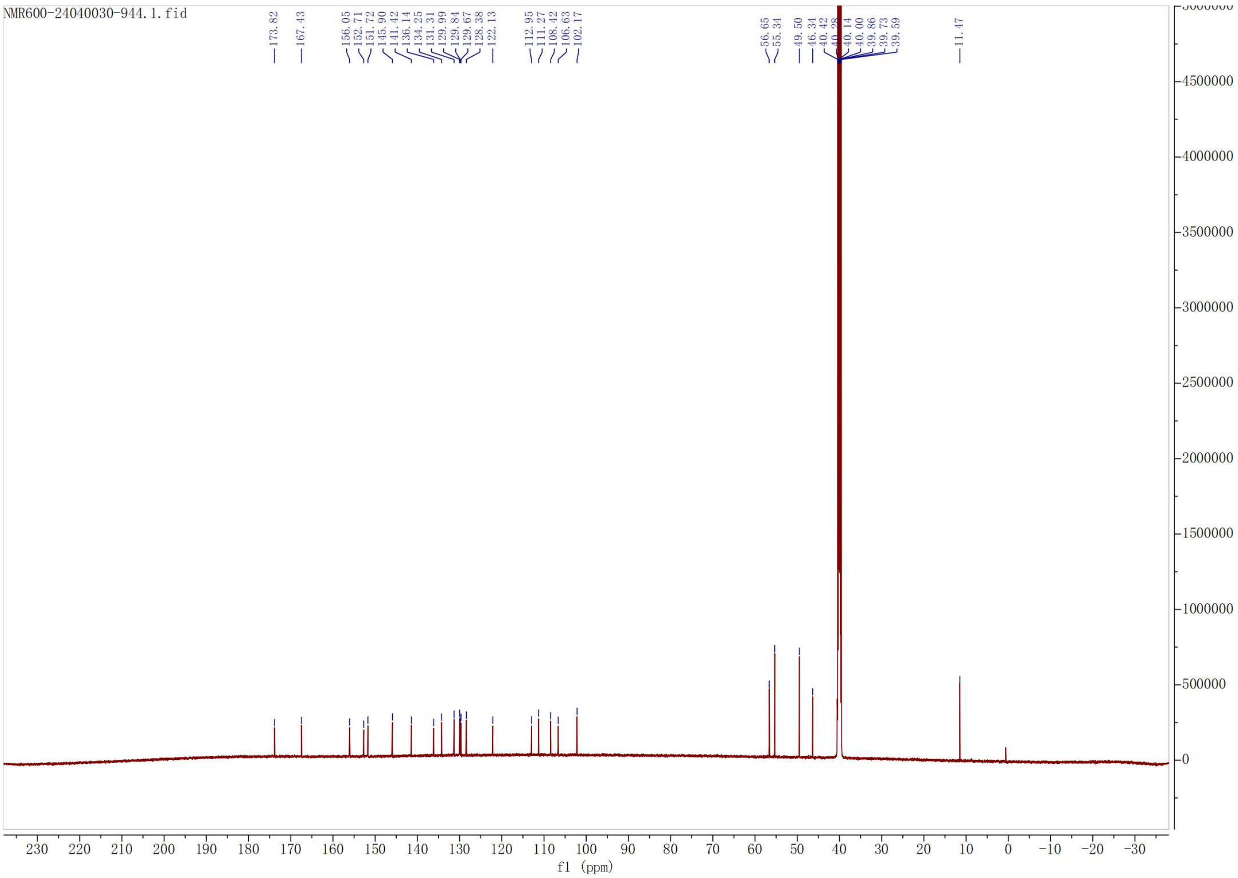
NMR600-24040030-944.1.fid
173.82
167.43
156.05
152.71
151.72
145.90
141.42
136.14
134.25
131.31
129.99
129.84
129.67
128.38
122.13
112.95
111.27
108.42
106.63
102.17
56.65
55.34
49.50
46.34
40.42
40.28
40.14
40.00
39.86
39.73
39.59
11.47
f1 (ppm)

IIP0945

^{1}H NMR (400 MHz, DMSO-d_6) δ 8.34 (d, J = 1.2 Hz, 1 H), 8.17 (d, J = 0.8 Hz, 1 H), 7.83 (d, J = 1.2 Hz, 1 H), 7.76 (dd, J = 8.0, 1.6 Hz, 1 H), 7.65-7.59 (m, 2 H), 7.56-7.47 (m, 3 H), 6.82 (d, J = 8.8 Hz, 1 H), 3.73 (s, 3 H), 2.78 (t, J = 4.8 Hz, 4 H), 2.38 (t, J = 5.2 Hz, 4 H), 2.22 (s, 3 H), 2.03 (s, 3 H).

^{13}C NMR (151 MHz, CD_3SOCD_3) δ 166.74, 159.83, 158.29, 145.85, 145.73, 142.77, 133.97, 131.08, 129.98, 129.10, 126.87, 126.64, 124.51, 111.64, 109.96, 108.84, 108.08, 56.48, 55.27, 49.73, 46.30, 40.42, 40.28, 40.14, 40.00, 39.87, 39.73, 39.59, 12.04.

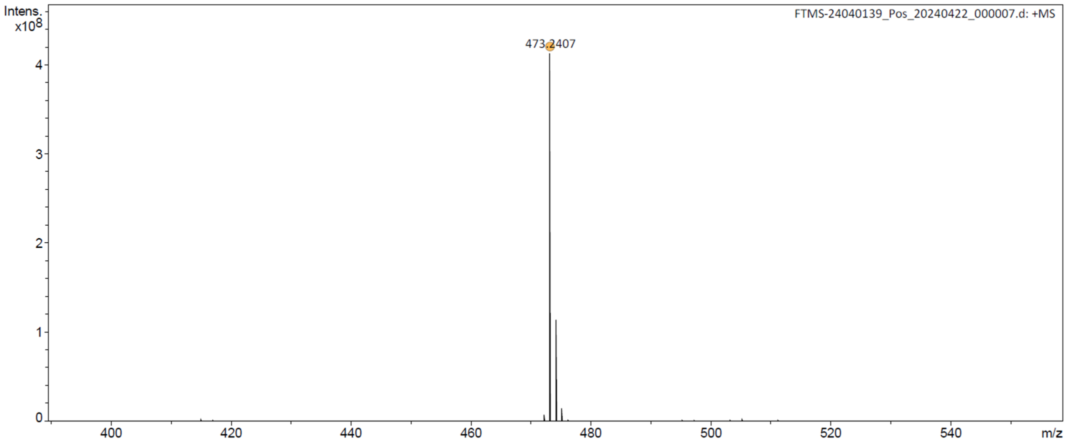

Meas. m/z	#	Ion Formula	Score	m/z	err [ppm]	Mean err [ppm]	mSigma	rdb	e^- Conf	N-Rule
473.2407	1	C25H29N8O2	100.00	473.2408	0.1	0.2	2.8	16.0	even	ok

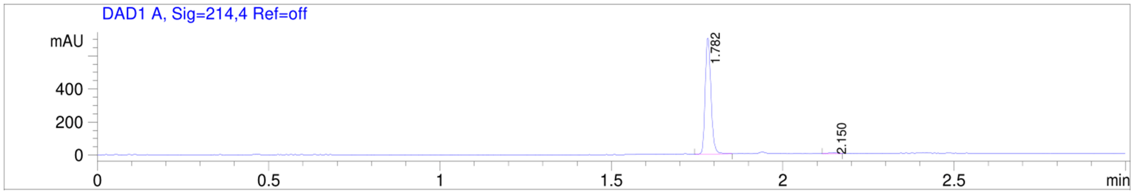

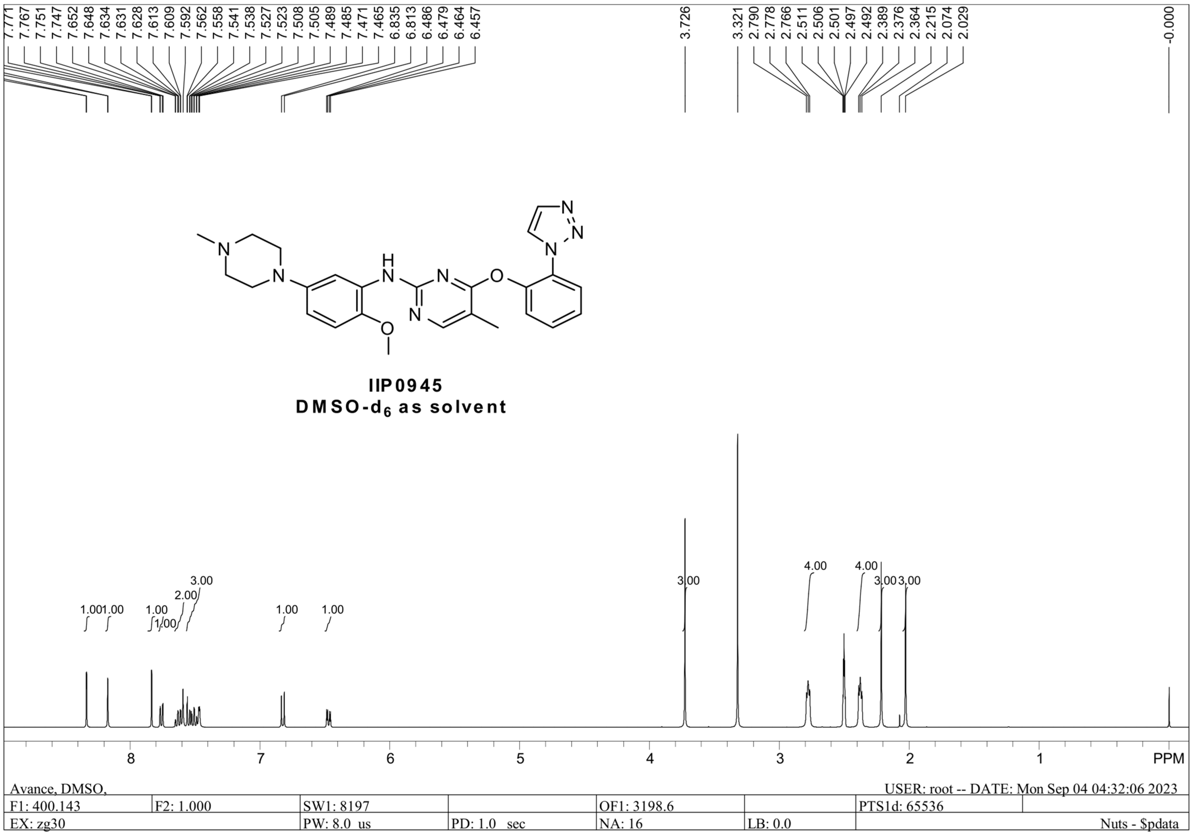

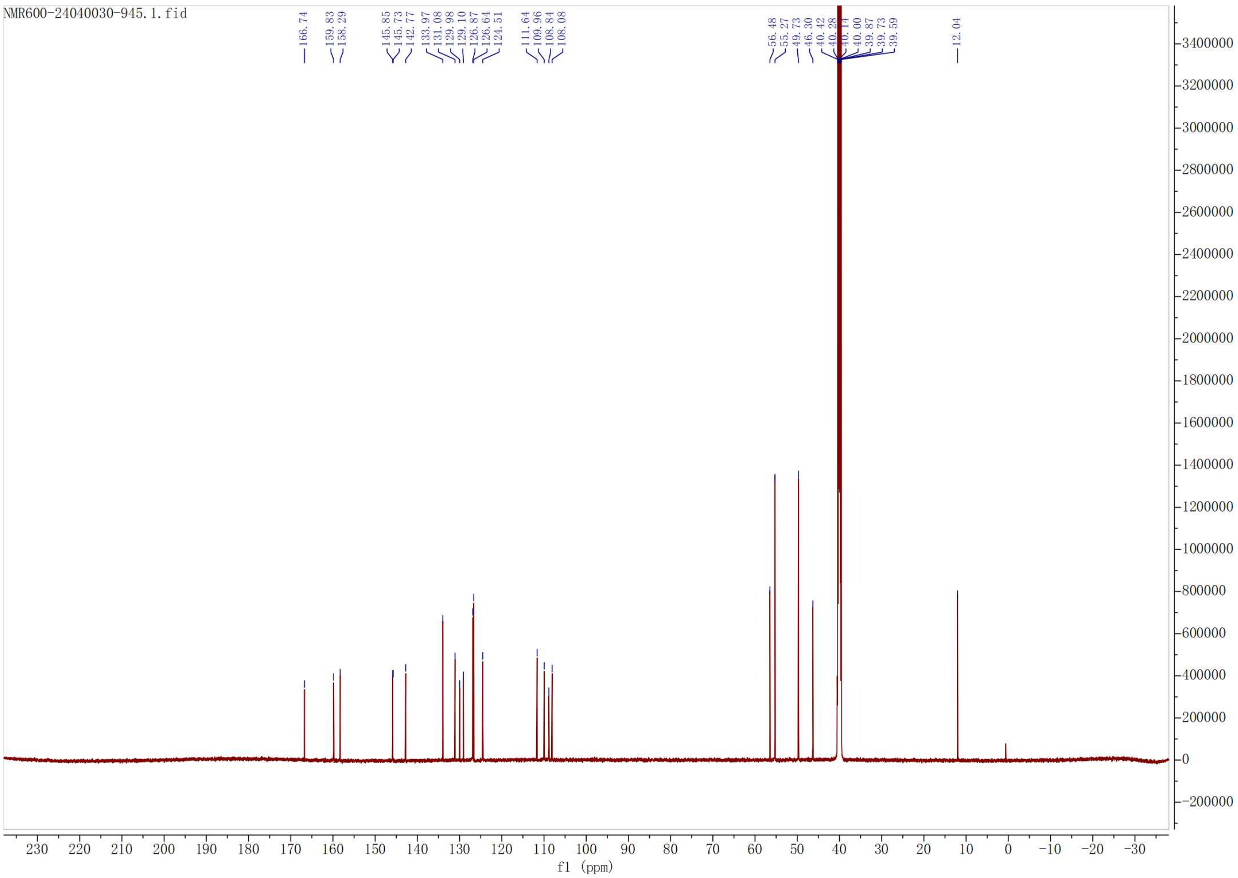
NMR600-24040030-945.1.fid
166.74
159.83
158.29
145.85
145.73
142.77
133.97
131.08
129.98
129.10
126.87
126.64
124.51
111.64
109.96
108.84
108.08
56.48
55.27
49.73
46.30
40.42
40.28
40.14
40.00
39.87
39.73
39.59
12.04
f1 (ppm)

Supplementary References

1. Brown, N., Fiscato, M., Segler, M. H. & Vaucher, A. C. GuacaMol: Benchmarking Models for de Novo Molecular Design. *Journal of Chemical Information and Modeling* **59.** PMID: 30887799, 1096–1108 (2019).
2. Gaulton, A. *et al.* The ChEMBL database in 2017. *Nucleic acids research* **45,** D945–D954 (2017).
3. Agarwal, P., Huckle, J., Newman, J. & Reid, D. L. Trends in small molecule drug properties: a developability molecule assessment perspective. *Drug Discovery Today,* 103366 (2022).
4. Yang, S.-Y. Pharmacophore modeling and applications in drug discovery: challenges and recent advances. *Drug discovery today* **15,** 444–450 (2010).
5. Rogers, D. & Hahn, M. Extended-connectivity fingerprints. *Journal of chemical information and modeling* **50,** 742–754 (2010).
6. Gillet, V. J., Willett, P. & Bradshaw, J. *Similarity searching using reduced graphs* in. **43** (Mar. 2003), 338–345. doi:10.1021/ci025592e.
7. Barker, E. J., Gardiner, E. J., Gillet, V. J., Kitts, P. & Morris, J. *Further development of reduced graphs for identifying bioactive compounds* in. **43** (Mar. 2003), 346–356. doi:10.1021/ci0255937.
8. Sun, J. *et al.* ExCAPE-DB: an integrated large scale dataset facilitating Big Data analysis in chemogenomics. *Journal of cheminformatics* **9,** 1–9 (2017).
9. Pogány, P., Arad, N., Genway, S. & Pickett, S. D. De novo molecule design by translating from reduced graphs to SMILES. *Journal of chemical information and modeling* **59,** 1136–1146 (2018).
10. Polykovskiy, D. *et al.* Molecular sets (MOSES): a benchmarking platform for molecular generation models. *Frontiers in pharmacology* **11,** 565644 (2020).
11. Olivecrona, M., Blaschke, T., Engkvist, O. & Chen, H. Molecular de-novo design through deep reinforcement learning. *Journal of cheminformatics* **9,** 1–14 (2017).
12. Blaschke, T. *et al.* REINVENT 2.0: an AI tool for de novo drug design. *Journal of chemical information and modeling* **60,** 5918–5922 (2020).
13. Caruso, M. *et al.* 5-(2-amino-pyrimidin-4-yl)-1H-pyrrole and 2-(2-amino-pyrimidin-4-yl)-1, 5, 6, 7-tetrahydro-pyrrolo [3, 2-c] pyridin-4-one derivatives as new classes of selective and orally available Polo-like kinase 1 inhibitors. *Bioorganic & medicinal chemistry letters* **22,** 96–101 (2012).
14. Beria, I. *et al.* Identification of 4, 5-Dihydro-1 H-pyrazolo [4, 3-H] quinazoline derivatives as a new class of orally and selective polo-like kinase 1 inhibitors. *Journal of medicinal chemistry* **53,** 3532–3551 (2010).
15. Zhou, W. *et al.* Novel mutant-selective EGFR kinase inhibitors against EGFR T790M. *Nature* **462,** 1070–1074 (2009).
16. Romu, A. A., Lei, Z., Zhou, B., Chen, Z.-S. & Korlipara, V. Design, synthesis and biological evaluation of WZ4002 analogues as EGFR inhibitors. *Bioorganic & Medicinal Chemistry Letters* **27,** 4832–4837 (2017).
17. Stadtmueller, H. & Sapountzis, I. *Substituted pyrimidines for the treatment of diseases such as cancer* U.S. Patent 8,846,689 B2, Sep. 30, 2014.
18. Popow, J. *et al.* Highly selective PTK2 proteolysis targeting chimeras to probe focal adhesion kinase scaffolding functions. *Journal of Medicinal Chemistry* **62,** 2508–2520 (2019).
19. Landrum, G. A., Penzotti, J. E. & Putta, S. Feature-map vectors: a new class of informative descriptors for computational drug discovery. *Journal of computer-aided molecular design* **20,** 751–762 (2006).
