# Supplementary material for: Accelerating discovery of bioactive ligands with pharmacophore-informed generative models
Source: Nat Commun. 2025 Mar 10;16:2391. doi: 10.1038/s41467-025-56349-0 (PMC11894060; doi:10.1038/s41467-025-56349-0)
Supplement: Supplementary file 2 — Reporting Summary [file 41467_2025_56349_MOESM2_ESM.pdf]

nature portfolio

Corresponding author(s): Jianfeng Pei

Last updated by author(s): Nov 28, 2024

Reporting Summary

Nature Portfolio wishes to improve the reproducibility of the work that we publish. This form provides structure for consistency and transparency in reporting. For further information on Nature Portfolio policies, see our Editorial Policies and the Editorial Policy Checklist.

Statistics

For all statistical analyses, confirm that the following items are present in the figure legend, table legend, main text, or Methods section.

n/a	Confirmed	
☐	☒	The exact sample size (*n*) for each experimental group/condition, given as a discrete number and unit of measurement
☐	☒	A statement on whether measurements were taken from distinct samples or whether the same sample was measured repeatedly
☒	☐	The statistical test(s) used AND whether they are one- or two-sided<br>*Only common tests should be described solely by name; describe more complex techniques in the Methods section.*
☒	☐	A description of all covariates tested
☒	☐	A description of any assumptions or corrections, such as tests of normality and adjustment for multiple comparisons
☐	☒	A full description of the statistical parameters including central tendency (e.g. means) or other basic estimates (e.g. regression coefficient) AND variation (e.g. standard deviation) or associated estimates of uncertainty (e.g. confidence intervals)
☐	☒	For null hypothesis testing, the test statistic (e.g. *F*, *t*, *r*) with confidence intervals, effect sizes, degrees of freedom and *P* value noted<br>*Give P values as exact values whenever suitable.*
☒	☐	For Bayesian analysis, information on the choice of priors and Markov chain Monte Carlo settings
☒	☐	For hierarchical and complex designs, identification of the appropriate level for tests and full reporting of outcomes
☐	☒	Estimates of effect sizes (e.g. Cohen's *d*, Pearson's *r*), indicating how they were calculated

Our web collection on statistics for biologists contains articles on many of the points above.

Software and code

Policy information about availability of computer code

Data collection	Custom codes used in this study are available at https://github.com/iipharma/transpharmer-repo.
Data analysis	Glide v7.8 was used to perform molecular docking. Gromacs 2020 was used to perform MD simulations. gmx_MMPBSA 1.6 was used to estimate binding free energy. PLIP <2.3 was used to analyze protein-ligand interactions.

For manuscripts utilizing custom algorithms or software that are central to the research but not yet described in published literature, software must be made available to editors and reviewers. We strongly encourage code deposition in a community repository (e.g. GitHub). See the Nature Portfolio guidelines for submitting code & software for further information.

Data

Policy information about availability of data

All manuscripts must include a data availability statement. This statement should provide the following information, where applicable:

- Accession codes, unique identifiers, or web links for publicly available datasets
- A description of any restrictions on data availability
- For clinical datasets or third party data, please ensure that the statement adheres to our policy

Source data are provided with this paper as Source Data file. Supplementary data in this study are provided in the Supplementary Information. The generated molecules in the case studies of DRD2 and PLK1 in this study have been deposited in the Zenodo database. The crystal structure of PLK1 used in this study are available in the RCSB PDB database under accession code 2YAC [https://doi.org/10.2210/pdb2YAC/pdb].

Research involving human participants, their data, or biological material

Policy information about studies with human participants or human data. See also policy information about sex, gender (identity/presentation), and sexual orientation and race, ethnicity and racism.

Reporting on sex and gender	no human research participants information were used for this study.
Reporting on race, ethnicity, or other socially relevant groupings	no human research participants information were used for this study.
Population characteristics	no human research participants information were used for this study.
Recruitment	no human research participants information were used for this study.
Ethics oversight	no human research participants information were used for this study.

Note that full information on the approval of the study protocol must also be provided in the manuscript.

Field-specific reporting

Please select the one below that is the best fit for your research. If you are not sure, read the appropriate sections before making your selection.

☒ Life sciences ☐ Behavioural & social sciences ☐ Ecological, evolutionary & environmental sciences

For a reference copy of the document with all sections, see nature.com/documents/nr-reporting-summary-flat.pdf

Life sciences study design

All studies must disclose on these points even when the disclosure is negative.

Sample size	Sample size estimation was not relevant for this study. For the enzyme and cell assays, three samples were included to meet the requirements for statistical analysis.
Data exclusions	no data were excluded
Replication	The computational random number is seeded for reproducibility. For the enzyme and cell assays, each assay was performed at least three times to confirm the results and these replicates were used to calculate mean values and standard deviations.
Randomization	For all in vitro assays, samples were randomized into different control/treatment groups.
Blinding	For in vitro experiments, blinding was not possible because researchers were involved in induction procedures.

Reporting for specific materials, systems and methods

We require information from authors about some types of materials, experimental systems and methods used in many studies. Here, indicate whether each material, system or method listed is relevant to your study. If you are not sure if a list item applies to your research, read the appropriate section before selecting a response.

Materials & experimental systems

n/a	Involved in the study	
☒	☐	Antibodies
☐	☒	Eukaryotic cell lines
☒	☐	Palaeontology and archaeology
☒	☐	Animals and other organisms
☒	☐	Clinical data
☒	☐	Dual use research of concern
☒	☐	Plants

Methods

n/a	Involved in the study	
☒	☐	ChIP-seq
☒	☐	Flow cytometry
☒	☐	MRI-based neuroimaging

Eukaryotic cell lines

Policy information about cell lines and Sex and Gender in Research

Cell line source(s)	HCT116 cell line is provided by Procell Life Science&Technology Co., Ltd.
Authentication	The cell line used is commercially available and not authenticated.
Mycoplasma contamination	cell lines tested were negative for mycoplasma contamination
Commonly misidentified lines (See ICLAC register)	no commonly misidentified lines were used

Plants

Seed stocks	*Report on the source of all seed stocks or other plant material used. If applicable, state the seed stock centre and catalogue number. If plant specimens were collected from the field, describe the collection location, date and sampling procedures.*
Novel plant genotypes	*Describe the methods by which all novel plant genotypes were produced. This includes those generated by transgenic approaches, gene editing, chemical/radiation-based mutagenesis and hybridization. For transgenic lines, describe the transformation method, the number of independent lines analyzed and the generation upon which experiments were performed. For gene-edited lines, describe the editor used, the endogenous sequence targeted for editing, the targeting guide RNA sequence (if applicable) and how the editor was applied.*
Authentication	*Describe any authentication procedures for each seed stock used or novel genotype generated. Describe any experiments used to assess the effect of a mutation and, where applicable, how potential secondary effects (e.g. second site T-DNA insertions, mosiacism, off-target gene editing) were examined.*
